# Insights into the Influence of Membrane Permeability and Structure on Osmotically-Driven Membrane Processes

**DOI:** 10.3390/membranes11020153

**Published:** 2021-02-22

**Authors:** Jing Wei, Qianhong She, Xin Liu

**Affiliations:** 1 School of the Environment and Safety Engineering, Jiangsu University, 301 Xuefu Road, Zhenjiang 212013, Jiangsu, China; weijing@ujs.edu.cn; 2 Institute of Environmental Health and Ecological Security, Jiangsu University, 301 Xuefu Road, Zhenjiang 212013, Jiangsu, China; 3 Singapore Membrane Technology Centre, Nanyang Technological University, 1 Cleantech Loop, Singapore 637141, Singapore; liux5@sustc.edu.cn; 4 School of Civil and Environmental Engineering, Nanyang Technological University, 50 Nanyang Avenue, Singapore 639798, Singapore

**Keywords:** osmotically-driven membrane process, internal concentration polarization, forward osmosis efficiency, permeability, structure

## Abstract

The success of osmotically-driven membrane (OM) technology relies critically on high-performance membranes. Yet trade-off of membrane properties, often further complicated by the strongly non-linear dependence of OM performance on them, imposes important constraint on membrane performance. This work systematically characterized four typical commercial osmotic membranes in terms of intrinsic separation parameters, structure and surface properties. The osmotic separation performance and membrane scaling behavior of these membranes were evaluated to elucidate the interrelationship of these properties. Experimental results revealed that membranes with smaller structural parameter (*S*) and higher water/solute selectivity underwent lower internal concentration polarization (ICP) and exhibited higher forward osmosis (FO) efficiency (i.e., higher ratio of experimental water flux over theoretical water flux). Under the condition with low ICP, membrane water permeability (*A*) had dominant effect on water flux. In this case, the investigated thin film composite membrane (TFC, *A* = 2.56 L/(m^2^ h bar), *S* = 1.14 mm) achieved a water flux up to 82% higher than that of the asymmetric cellulose triacetate membrane (CTA-W(P), *A* = 1.06 L/(m^2^ h bar), *S* = 0.73 mm). In contrast, water flux became less dependent on the *A* value but was affected more by membrane structure under the condition with severe ICP, and the membrane exhibited lower FO efficiency. The ratio of water flux (*J_v TFC_*/*J_v CTA-W(P)_*) decreased to 0.55 when 0.5 M NaCl feed solution and 2 M NaCl draw solution were used. A framework was proposed to evaluate the governing factors under different conditions and to provide insights into the membrane optimization for targeted OM applications.

## 1. Introduction

**Figure 1:**
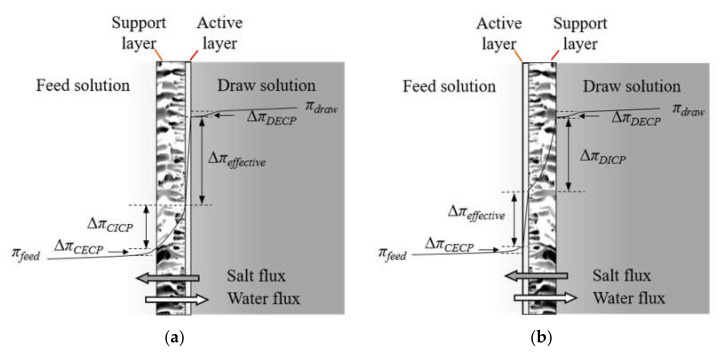
Schematic illustration of osmotic pressure profile across the membrane in osmotically-driven membrane processes. (**a**) Active-layer-facing-draw-solution (AL-DS) mode. (**b**) Active-layer-facing-feed-solution (AL-FS) mode. Δ*π_effective_* is the effective osmotic pressure difference. Δ*π_CICP_*, Δ*π_DICP_*, Δ*π_CECP_*, and Δ*π_DECP_* are the osmotic pressure difference loss caused by concentrative internal concentration polarization (ICP), dilutive ICP, concentrative external concentration polarization (ECP), and dilutive ECP [18].

Osmotically-driven membrane (OM) technologies, e.g., forward osmosis (FO) and pressure-retarded osmosis (PRO), have received much attention, largely fueled by the development of commercial FO membranes with high water flux in the last decade. In an OM process, a semi-permeable membrane is used to separate a high concentration solution (draw solution, DS) and a low concentration solution (feed solution, FS) (Figure 1). Water flux from FS to DS is driven by the osmotic pressure difference (Δ*π*). Components in FS can be retained and concentrated, whereas water passing through the membrane can be recovered from the diluted DS by a re-concentration step if required. Niche applications using DSs without the need of regeneration (e.g., seawater [1], desalination brine [2], or fertilizer solutions [3]) are attractive due to the elimination of energy-intensive DS re-concentration step. OM and hybrid OM processes have been explored for diverse fields [4], such as wastewater reuse [5,6,7], desalination [8,9,10,11], osmotic membrane bioreactor [12], energy production [13,14,15], and food processing [16,17].

High-performance membranes are crucial for OM technologies. Typical osmotic membranes consist of an active rejection layer and a porous support layer. The support layer, usually orders of magnitude thicker than the ultrathin active layer, hinders the mass transfer within membrane and causes internal concentration polarization (ICP) (Figure 1). Performance of membranes are often limited by ICP that reduces the effective osmotic pressure difference across the active layer [19,20]. Concentrative ICP of feed solutes occurs in the active-layer-facing-draw-solution (AL-DS) orientation, and dilutive ICP of draw solutes occurs in the active-layer-facing-feed-solution (AL-FS) orientation [21]. If the membranes have low rejection, reverse diffusion of draw solutes into the FS can further enhance ICP [22]. Due to the ICP phenomenon, membranes with a compact support layer (e.g., those used for reverse osmosis (RO) and nanofiltration (NF)) usually exhibit very low water flux in OM tests.

Some promising membranes for OM process have been developed in recent years. Membranes for FO processes were first commercialized by Hydration Technology Innovations (HTI, formerly Osmotek, Albany, OR, USA) [23]. Their first generation of membranes were flat-sheet cellulose triacetate (CTA) integral asymmetric membranes [24]. These membranes showed superior FO water flux compared to commercial RO membranes, and they have been widely used in OM study. CTA hollow fiber FO and PRO membranes have been developed by Toyobo (Osaka, Japan) [25,26]. Commercial thin film composite (TFC) FO membranes were also provided by a few companies, including HTI [27], Oasys Water (Boston, MA, USA) [28], Toray Chemical Korea (Seoul, Korea) [29], Woongjin Chemical (Seoul, Korea) [30], Porifera Inc. (San Leandro, CA, USA) [31], Aquaporin (Kongens Lyngby, Denmark) [32,33], etc. In parallel, considerable studies of lab-scale membrane fabrication have been reported [34,35,36]. These membranes had different structures, e.g., integral asymmetric membrane [37,38], TFC membrane [39,40,41], double-skinned membrane [42], dual-layer membrane [43], etc. According to the water and salt permeability, these membranes can be classified into RO-like [39,40], NF-like [22,44,45], and ultrafiltration (UF)-like [46] membranes. Generally, the criteria of high-performance OM membranes include high water permeability (*A*), low solute permeability (*B*), and small structural parameter (*S*), as well as other properties, such as fouling resistance and mechanical strength. Nevertheless, improvement of OM performance is constrained by the strong trade-off among these parameters [47] (e.g., *A* and *B*), and it is further complicated by its non-linear dependence on membrane properties and FS/DS concentrations [22]. Consequently, membrane optimization and selection shall be based on a set of comprehensive criteria, as well as understanding of the dominant mechanisms that govern the OM performance.

The objectives of this work were to study the influence of membrane permeability and structure on OM performance and to develop efficient strategies for OM flux enhancement. Commercial FO membranes with different structure, separation property and chemical property were systematically characterized to study the trade-off of key membrane properties. According to the FS and DS sources in potential applications, a series of OM experiments were conducted to assess the advantages and drawbacks of these membranes. The OM performance especially the water flux and FO efficiency of membranes were evaluated and compared. Membranes reported in the FO literature were also investigated to validate the comprehensive influence of membrane permeability and structure on water flux. Based on this study, a framework was developed to identify the factors that govern OM flux under different testing conditions, and to provide implications for the evaluation and optimization of membranes in OM processes.

## 2. Materials and Methods

### 2.1. Membranes and Chemicals

Four flat-sheet FO membranes, i.e., TFC, CTA-W(P), CTA-W, and CTA-NW were received from Hydration Technology Innovations (HTI, Albany, OR, USA), which are still commercially available from Fluid Technology Solutions, Inc. (FTSH_2_O, Albany, OR, USA) [48]. According to the manufacturer, the thin film composite membrane (referred to as TFC) had a mesh embedded in the substrate. Two of the integral asymmetric membranes (referred to as CTA-W(P) and CTA-W) had an embedded woven mesh. The integral asymmetric membrane with a nonwoven fabric was referred to as CTA-NW. All the membranes were stored in pure water before characterization.

Sodium chloride (NaCl), sodium sulfate (Na_2_SO_4_) and calcium chloride (CaCl_2_) for OM tests were purchased from Merck (Darmstadt, Germany). All chemicals were analytical grade and were used as received. Ultrapure water with resistivity of 18.2 MΩ·cm was supplied by a Milli-Q water system (Milli-pore, Darmstadt, Germany).

### 2.2. Membrane Characterization

#### 2.2.1. Characterization of Membrane Structure, Morphology and Chemical Property

Membrane structure and morphology were observed by field-emission scanning electron microscopy (FESEM, JSM-7600F, JEOL, Akishima, Japan). The membranes were freeze-dried before characterization. Samples were sputter coated a thin layer of platinum before FESEM scanning. The images were obtained with secondary electrons at 5.0 kV. Chemical compositions of membranes were characterized by Fourier transform infrared spectroscopy (FTIR, Spectrum 2000 FTIR Spectrometer, PerkinElmer, Boston, MA, USA) in attenuated total reflectance (ATR) mode. Porosity of membranes was evaluated by gravimetric method. Membrane samples were saturated with water and then thoroughly dried. The wet mass and dry mass were measured to calculate the porosity in accordance to previous work [24]. Surface roughness of membranes was characterized by atomic force microscopy (AFM, XE-100, Park Systems, Suwon, Korea). The root mean squared roughness (*R_q_*) of membrane rejection layer was reported. Water contact angles of membranes were measured by static sessile drop method using a contact angle system (OCA, Dataphysics, Filderstadt, Germany). Mechanical strength of membranes was measured using a tensile testing system (Instron 5567, Norwood, MA, USA). For membranes with a mesh as reinforcing layer (TFC, CTA-W(P), and CTA-W), tensile testing was performed with tension in both the axial direction along filaments and the diagonal direction of mesh opening.

#### 2.2.2. Measurement of Membrane Permeability

The water and salt permeabilities of membranes were determined in a cross-flow RO filtration setup (Figure S1). The temperature of feed was maintained at 23 ± 1 °C and the feed pressure was 5 bar for all tests. Water permeability coefficient (*A*) was measured using pure water as feed. To measure the salt rejection of membrane, 10 mM NaCl or 10 mM Na_2_SO_4_ solutions were used as feeds. The concentrations of feed and permeate were measured using a conductivity meter (UltraMeter II™ 4P, Myron L Company, Carlsbad, CA, USA) to calculate the salt rejection (*R*) and salt permeability coefficient (*B*) [22]. The *A*, *R*, and *B* values of membranes were calculated according to:

(1) $$A=\frac{J}{\Delta P},$$

(2) $$R=\frac{C_{f}-C_{p}}{C_{f}}\times 100\%,$$

(3) $$B=\left(\frac{1}{R}-1\right)\times J,$$

where *J* is the flux of permeate; Δ*P* is the transmembrane pressure; *C_f_* and *C_p_* are the salt concentrations of feed and permeate, respectively.

#### 2.2.3. Evaluation of OM Performance

FO performance of membranes were evaluated in a cross-flow FO setup in both AL-DS and AL-FS orientations in accordance to previous studies [22,24]. NaCl solutions with concentrations of 0, 10 mM, and 0.5 M were used as FS to simulate pure water, freshwater and seawater. NaCl solutions with concentrations of 0.2 M, 0.5 M, and 2 M were used as DS to simulate brackish water, seawater, and high-salinity brine. A cross-flow velocity of 23.2 cm/s was applied to both FS and DS channels. Water flux (*J_v_*) and salt flux (*J_s_*) was calculated as the permeate rate of water and salt per unit of membrane area [22,24]. FO efficiency (*J_v_*/*J_v Theo_*) was calculated as the ratio of experimental FO water flux over the theoretical water flux (*J_v Theo_* = *A*Δ*π*) that can be achieved when there was no concentration polarization. Mass transfer coefficient (*K_m_*) and structural parameter (*S*) of membranes were calculated according to the ICP model [49], as follows:

(4) $$\mathrm{AL}-\mathrm{DS}:\;J_{v}=K_{m}ln\frac{\pi_{draw}-\frac{J_{v}}{A}+\frac{B}{A}}{\pi_{feed}+\frac{B}{A}},$$

(5) $$\mathrm{AL}-\mathrm{FS}:\;J_{v}=K_{m}ln\frac{\pi_{draw}+\frac{B}{A}}{\pi_{feed}+\frac{J_{v}}{A}+\frac{B}{A}},$$

(6) $$K_{m}=\frac{D}{S},$$

where *π_draw_* and *π_feed_* are the osmotic pressures of DS and FS, respectively; *D* is the diffusion coefficient of solute.

FO scaling experiments were conducted using gypsum as a model scalant. The tests were performed in the AL-FS mode. This mode is preferred in FO applications with low scaling/fouling potential as it can eliminate the internal clogging of support layer and achieve more stable flux in long-term operation [50]. In the scaling tests, mixed solution containing Na_2_SO_4_ (72 mM), CaCl_2_ (26.1 mM), and NaCl (10 mM) was used as FS, of which the gypsum saturation index (SI) was 2.0 [51]. Baseline tests were performed using 0.163 M NaCl FS, of which the SI was 0 and the osmotic pressure (7.5 bar) was similar with that of scaling test. DS with concentration of 2–3 M NaCl was used to adjust initial *J_v_* to a moderate value of 15 L/(m^2^ h) for each test. A cross-flow velocity of 23.2 cm/s was applied to FS and DS channels.

PRO tests were performed in a cross-flow PRO setup as in reference [52]. Membrane coupons were loaded in membrane cell with AL-DS orientation. Net-type spacers were placed on both the FS and DS sides of membrane. 1 M NaCl was used as DS to simulate seawater desalination brine, and was circulated by a high-pressure pump, while 10 mM NaCl FS was circulated without pressurization. Draw pressure (*P_draw_*) was increased in steps of approximately 3 bar, until the peak power density was reached. Pressure at FS side (*P_feed_*) was also monitored to calculate the pressure difference (Δ*P* = *P_draw_* − *P_feed_*). During PRO tests, pure water was dosed into FS to maintain a constant feed volume. *J_v_* and *J_s_* at each pressure were measured as the water and salt permeate rate per unit of membrane area. Power density (*W*) of membrane was calculated as:

(7) $$W=J_{v}\times \Delta P.$$

## 3. Results and Discussions

### 3.1. Structure, Morphology, and Chemical Property of Membranes

**Figure 2:**
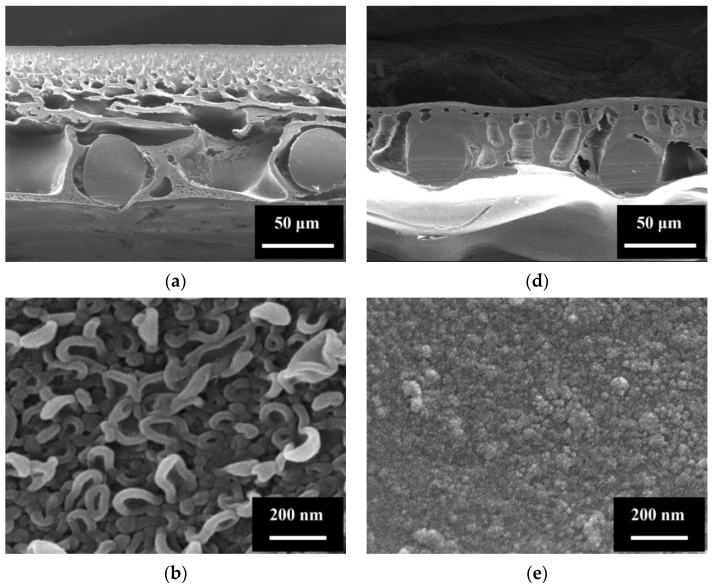

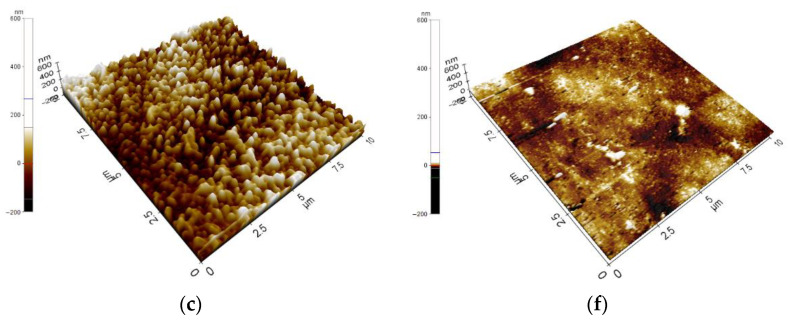
Structure and surface morphology of the thin film composite membrane (TFC) and cellulose triacetate membrane (CTA-W(P)). (**a**) Field-emission scanning electron microscopy (FESEM) micrograph of TFC cross-section at 500×. (**b**) FESEM micrograph of the surface of TFC active layer at 100,000×. (**c**) Atomic force microscopy (AFM) image of the surface of TFC active layer. (**d**) FESEM micrograph of CTA-W(P) cross-section at 500×. (**e**) FESEM micrograph of the surface of CTA-W(P) active layer at 100,000×. (**f**) AFM image of the surface of CTA-W(P) active layer.

**Figure 3:**
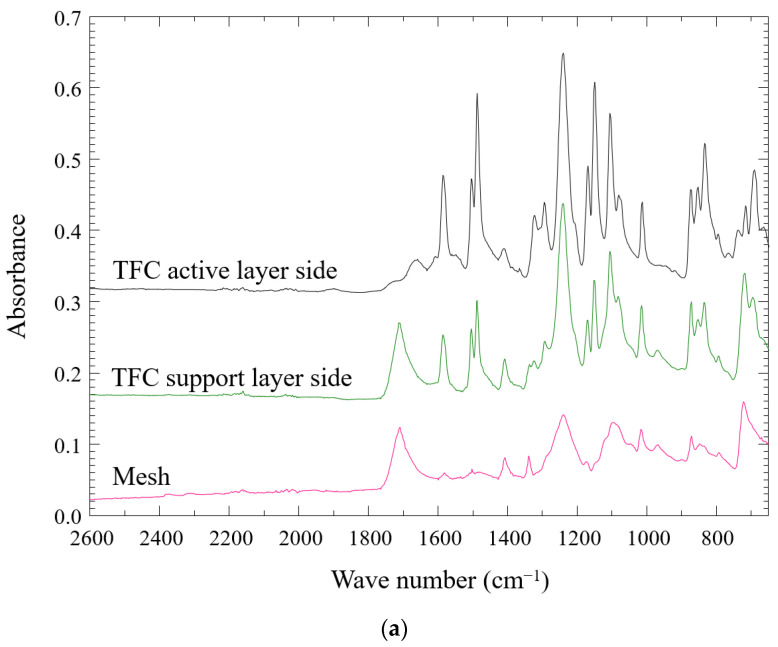

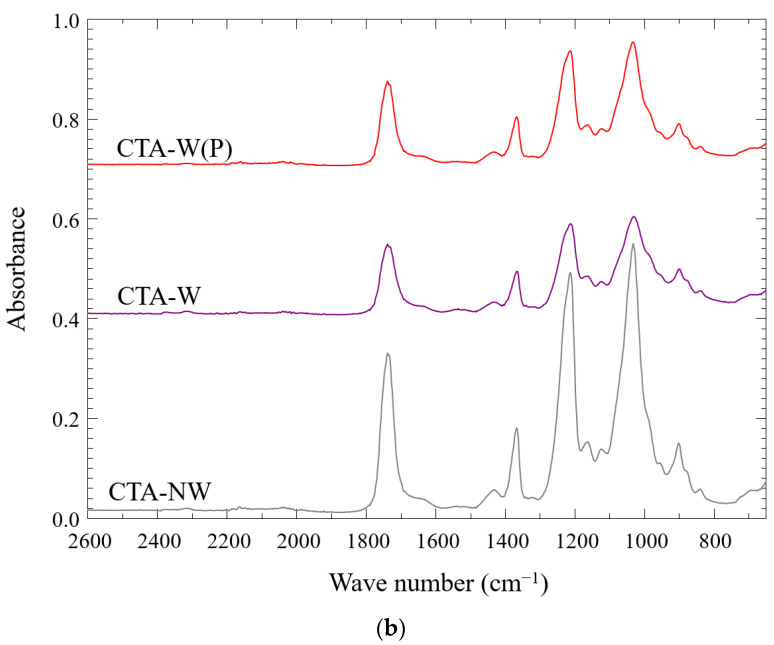
Fourier transform infrared spectroscopy (FTIR) spectra of (**a**) TFC, and (**b**) CTA-W(P), CTA-woven (W), and CTA-nonwoven (NW) membranes.

Morphology of the membranes was characterized using FESEM and AFM. TFC had a three-layer structure (Figure 2a): (1) a polyamide (PA) top layer featured with a ridge-and-valley surface morphology (Figure 2b,c) and characteristic FTIR peaks at 1659 cm^−1^ (amide I band), 1609 cm^−1^ (aromatic amide) and 1549 cm^−1^ (amide II band) [53] (Figure 3a), (2) a polysulfone (PSf) support layer with finger-like pore structure, and (3) a polyester (PET) mesh embedded at the bottom of PSf layer for mechanical reinforcement. The thin and porous support layer (thickness of 105 μm and porosity of 66%, Table 1) resulted in a small *S* value (1.14 mm), which was an order of magnitude smaller than that of typical commercial TFC RO membranes [24].

**Figure A1:**
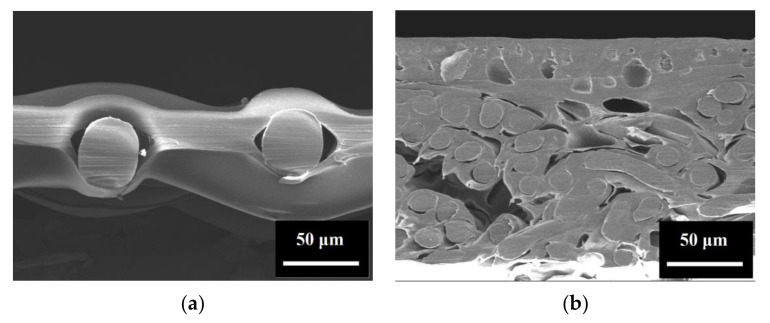
FESEM micrographs of the cross-sections (at 500×) of (**a**) CTA-W and (**b**) CTA-NW.

According to the FTIR results, CTA-W(P), CTA-W, and CTA-NW were all prepared with cellulose triacetate (Figure 3b). These membranes had smoother surfaces compared to the polyamide-based TFC. CTA-W(P) was an integral asymmetric membrane with a woven PET mesh embedded in the CTA layer (Figure 2d). Its CTA layer with finger-like pore structure had similar porosity (62%) but smaller thickness (52–71 μm) compared to TFC. Consequently, CTA-W(P) had a smaller *S* value of 0.73 mm. Structural property of CTA-W and CTA-NW had been reported in our previous study [24]. FESEM micrographs of their cross-sections can be found in Appendix A (Figure A1). CTA-W had a similar structure as CTA-W(P) with the same type of woven PET mesh embedded in support layer. However, CTA-W had a thinner and less porous CTA layer without macrovoid (Figure A1a). The *S* value of CTA-W (0.82 mm) was slightly higher than that of CTA-W(P). Different from CTA-W(P) and CTA-W, CTA-NW was reinforced by a nonwoven fabric attached at the bottom of CTA layer (Figure A1b). To mitigate ICP, the nonwoven fabric of CTA-NW was much looser compared to those used in commercial RO membranes. The CTA layer of CTA-NW had similar thickness (~60 μm) but less porous finger-like pore structure compared to CTA-W(P). Due to the large total membrane thickness (129 μm), CTA-NW had the largest *S* value (1.77 mm) of the four membranes.

**Figure A2:**
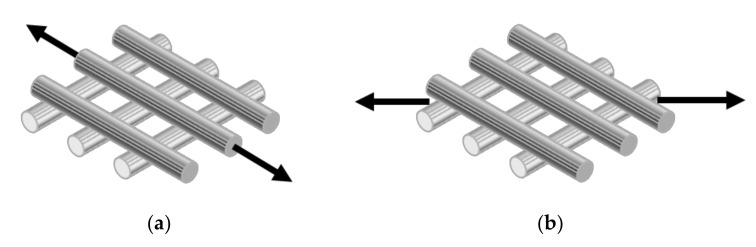
Illustration of tensile testing with tension in (**a**) axial direction and (**b**) diagonal direction.

Mechanical property of the membranes was characterized by tensile testing. Due to the porous structure, the mechanical strengths of PSf and CTA support layers were lower than the intrinsic values of bulk polymers. The membranes were strengthened by reinforcing fabric. The efficiency of fabric reinforcement depended on the orientation of fabric filaments. For membranes reinforced by plain woven mesh (TFC, CTA-W(P), and CTA-W), tensile tests were conducted at both axial and diagonal directions, as illustrated in Figure A2 in Appendix B. Membranes showed higher Young’s moduli and less strain at the point of break when tension was applied in the axial direction of mesh filaments (Table 1), implying a stronger reinforcement effect in this direction. For membrane reinforced by nonwoven fabric (CTA-NW), repeated measurements were conducted with tension at random directions. It showed isotropic mechanical property due to the randomly orientated filaments of nonwoven fabric. TFC membranes for pressure-driven process usually consist of a more compact nonwoven reinforcing fabric to reduce anisotropic stress and membrane deformation. The TFC FO membrane in this study showed compromised mechanical strength, as a result of the higher porosity and macrovoids in the PSf layer, as well as the mesh support (Figure 2a). Trade-off between mechanical strength and *S* value was also observed in the CTA membranes. For example, CTA-W with a dense support layer can stand higher stress at the point of break than CTA-W(P); however, it also resulted in a slightly larger *S* value.

### 3.2. Intrinsic Separation Property of Membranes

Water and salt permeabilities of membranes are shown in Table 1. The studied membranes had moderate to high rejections to NaCl and Na_2_SO_4_. CTA-W(P) had a water permeability of 1.06 L/(m^2^ h bar)), while lower water permeability of 0.41 L/(m^2^ h bar) and 0.53 L/(m^2^ h bar) were shown by CTA-W and CTA-NW, respectively. Compared to CTA-W(P), the dense structure of CTA-W was likely responsible for the increased hydraulic resistance. The low permeability of CTA-NW was mainly contributed by its thicker structure. Trade-off between membrane permeability and selectivity can be seen for the CTA membranes. Higher *A* value of CTA-W(P) was accompanied with a higher *B/A* value compared to CTA-W and CTA-NW. To overcome the limitation of CTA membrane, other types of membranes were also developed for OM process. Table A1 in Appendix C lists the intrinsic separation property of several commercial FO membranes reported. Most of these membranes were TFC membranes. Compared to the integral asymmetric CTA membranes, these TFC membranes had higher *A* values and smaller *B/A* values. As shown in Table 1, *A* value of TFC was 2.56 L/(m^2^ h bar), which was two times higher than that of CTA-W(P). The improvement could be attributed to the thinner and more hydrophilic polyamide layer (Table 1). Superior combination of water permeability and selectivity was also exhibited by TFC. *B_NaCl_/A* and *B_Na_*_2*SO*4_*/A* of TFC were 0.25 bar and 0.053 bar, respectively, both lower than those of CTA-W(P). As the salt flux/water flux (*J_s_/J_v_*) ratio is proportional to *B/A* value of membrane in OM process, the solute reverse diffusion of TFC would be less severe. Nevertheless, OM water flux does not linearly increase with membrane permeability and it also depends on the support layer. In this work, CTA-W(P) was used as a benchmark to comprehensively evaluate the influence of membrane property on OM performance.

### 3.3. Membrane Performance in Osmotically-Driven Processes

#### 3.3.1. FO Performance of Membranes

**Figure A3:**
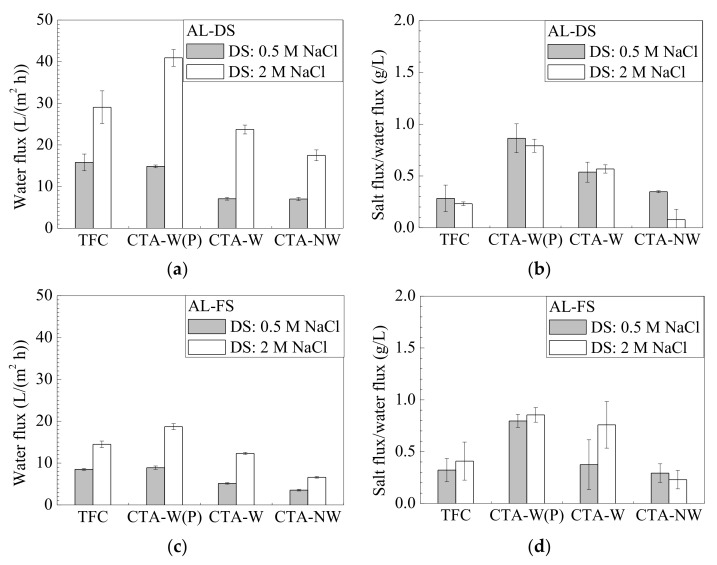
FO water flux and salt flux/water flux of the TFC, CTA-W(P), CTA-W, and CTA-NW membranes. Testing conditions: 10 mM NaCl as FS, and 0.5 M or 2 M NaCl as DS.

The FO performance of TFC, CTA-W(P), CTA-W and CTA-NW were evaluated using 0.5 M NaCl DS and 10 mM NaCl FS. In AL-DS mode, TFC had the highest *J_v_* and small *J_s_/J_v_* (Figure A3a,b in Appendix D). For the integral asymmetric CTA membranes, CTA-W and CTA-NW had relatively low *J_v_* that was approximately half of *J_v CTA-W(P)_*. It was mainly attributed to the low *A* values of CTA-W and CTA-NW, which were only half of *A_CTA-W(P)_*. CTA-W(P) also exhibited higher *J_v_* than CTA-W and CTA-NW when 2 M NaCl DS was used, in both AL-DS and AL-FS modes (Figure A3a,c). All the three CTA membranes in this study had moderate *J_s_/J_v_* (<1 g/L) (Figure A3b,d). In general, CTA-W(P) was more promising for OM applications than the other two CTA membranes due to its higher *A* value, smaller *S* value and moderate *B* value. Thus, CTA-W(P) was used for further OM study in this work.

**Figure 4:**
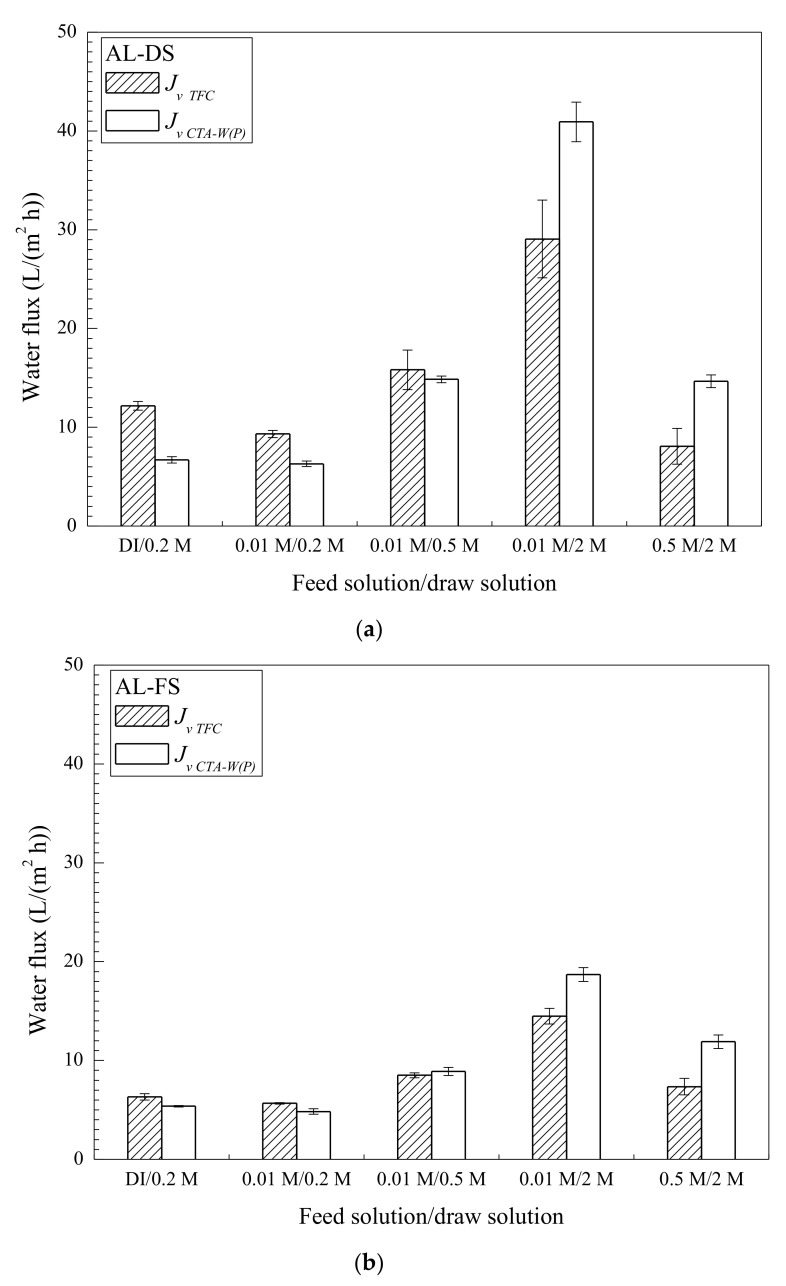
Forward osmosis (FO) water flux of TFC and CTA-W(P) membranes with different feed and draw solutions. The tests were performed in (**a**) AL-DS mode, and (**b**) AL-FS mode. Pure water, 10 mM and 0.5 M NaCl were used as feed solution (FS), which are denoted as DI, 0.01 M and 0.5 M. 0.2 M, 0.5 M, and 2 M NaCl were used as draw solution (DS), which are denoted as 0.2 M, 0.5 M, and 2 M.

As discussed in the above, TFC exhibited higher *J_v_* than CTA-W(P) in AL-DS mode when 0.5 M NaCl DS and 10 mM NaCl FS were used. However, when a more concentrated DS of 2 M NaCl was used, *J_v CTA-W(P)_* was about 30% higher than *J_v TFC_* in both AL-DS and AL-FS modes (Figure 4). In OM processes, *J_v_* is mainly governed by membrane frictional resistance loss, ICP, and solute reverse diffusion. Due to the low *J_s_/J_v_* of TFC and CTA-W(P), membrane frictional resistance loss and ICP played more important roles on *J_v_*. ICP was less severe when FS and DS concentrations were low, leading to a greater importance of *A* value for *J_v_*. Under such frictional-resistance-dominated conditions, the more permeable TFC achieved higher *J_v_*. When a more concentrated DS was used, however, ICP governed *J_v_* and CTA-W(P) with smaller *S* value performed better.

To further elucidate the governing mechanisms, forward osmosis (FO) tests with different FS and DS concentrations were conducted on TFC and CTA-W(P). *J_v_* of TFC and CTA-W(P) are compared in Figure 4. With a DI water FS and a low-concentration DS of 0.2 M NaCl (AL-DS), ICP was less severe, represented by a high FO efficiency of 0.70 for CTA-W(P). TFC achieved significantly higher *J_v_* than that of CTA-W(P) (*J_v TFC_/J_v CTA-W(P)_* = 1.82, Figure 4a). *J_v TFC_/J_v CTA-W(P)_* ratio was reduced as the concentrations of FS and DS increased. *J_v CTA-W(P)_* was higher than *J_v TFC_* when 2 M NaCl DS was used, especially when a concentrated FS of 0.5 M NaCl was used (*J_v TFC_/J_v CTA-W(P)_* = 0.55, AL-DS). This was attributed to the remarkably enhanced influence of ICP in this scenario, represented by a lower FO efficiency of 0.18 for CTA-W(P). In AL-FS mode, *J_v TFC_/J_v CTA-W(P)_* was 1.18 using 0.2 M NaCl DS and DI water FS (Figure 4b), which was significantly lower than *J_v TFC_/J_v CTA-W(P)_* in AL-DS mode. A low FO efficiency of 0.56 for CTA-W(P) implicated the more severe influence of dilutive ICP (AL-FS) than concentrative ICP (AL-DS) when identical FS and DS were used. Therefore, in AL-FS mode, more osmotic driving force was lost for TFC with larger *S* value. It can be seen from the above results that assessment of membranes in OM process should be based on membrane property and operational condition. Membranes with higher *J_v_* in one condition may show inferior *J_v_* in another condition. In view of the non-linear dependence of OM performance on membrane properties and the trade-off of these properties, their combined influences in different conditions will be further discussed.

#### 3.3.2. Anti-Scaling Performance of Membranes

**Figure 5:**
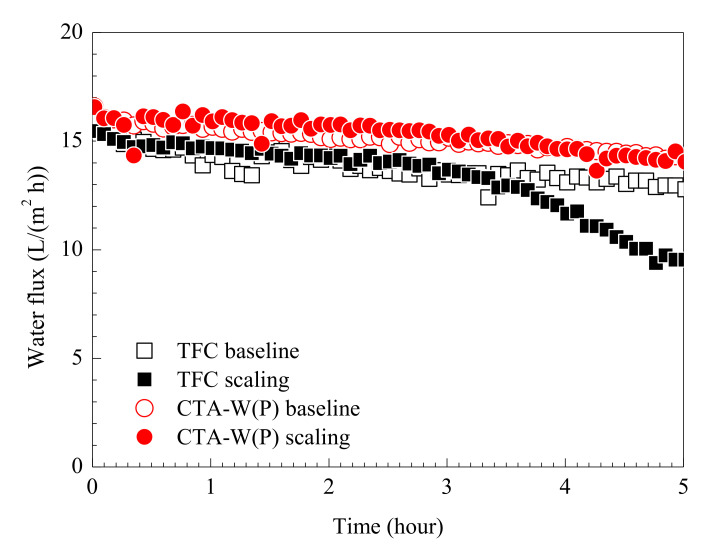
Water flux of TFC and CTA-W(P) in FO scaling tests. Testing conditions: FS for baseline tests was 0.163 M NaCl; FS for scaling tests was a mixed solution containing 72 mM Na_2_SO_4_, 26.1 mM CaCl_2_, and 10 mM NaCl; crossflow velocities of FS and DS on membrane were 23.2 cm/s; 2–3 M NaCl were used as DS to achieve initial water flux of 15 ± 1 L/(m^2^ h).

OM applications can use different types of saline water, which may contain sparingly soluble ions and impurities with high concentrations. For example, in desalination-hybrid processes where seawater or seawater desalination brine is used, a great amount of scaling precursors like Ca^2+^, Mg^2+^, SO_4_^2−^, and CO_3_^2−^ exist [54]. Sparingly soluble salts may precipitate and deposit on membrane during operation. In this study, FS containing Ca^2+^ and SO_4_^2−^ were used to investigate the impact of scaling on TFC and CTA-W(P). The membranes were tested in AL-FS mode with mitigated internal scaling [50]. Water flux of TFC and CTA-W(P) in the scaling tests are shown in Figure 5. CTA-W(P) exhibited stable performance during the scaling test of 18 h, without significant variation of *J_v_* compared to the baseline. In contrast, *J_v_* of TFC drastically declined after 3 h of scaling test. Severe leakage of draw solutes into FS took place after test of 5 h and resulted in the interruption of operation. Damage at the polyamide layer was observed when the tested membrane was later examined under FESEM (Figure S2). In the scaling test, Ca^2+^ and SO_4_^2−^ in FS were concentrated and caused precipitation of gypsum crystals. According to the FESEM and AFM images of membrane rejection layer (Figure 2), TFC had a rougher surface (roughness *R_q_* = 69 nm) than that of CTA-W(P) (*R_q_* = 5 nm) (Table 1). Moreover, the carboxylic groups of polyamide layer provided binding site for Ca^2+^ and led to surface crystallization of gypsum [55]. Therefore, faster deposition and growth of gypsum crystals were prone to take place on TFC. Crystallization beneath feed spacer filaments could also cause damage to membrane rejection layer, which tended to be more severe for the TFC membrane due to its thinner rejection layer [51]. Thus, CTA-W(P) had advantage in this condition because of its surface morphology, chemical property, and structure.

#### 3.3.3. PRO Performance of Membranes

**Figure 6:**
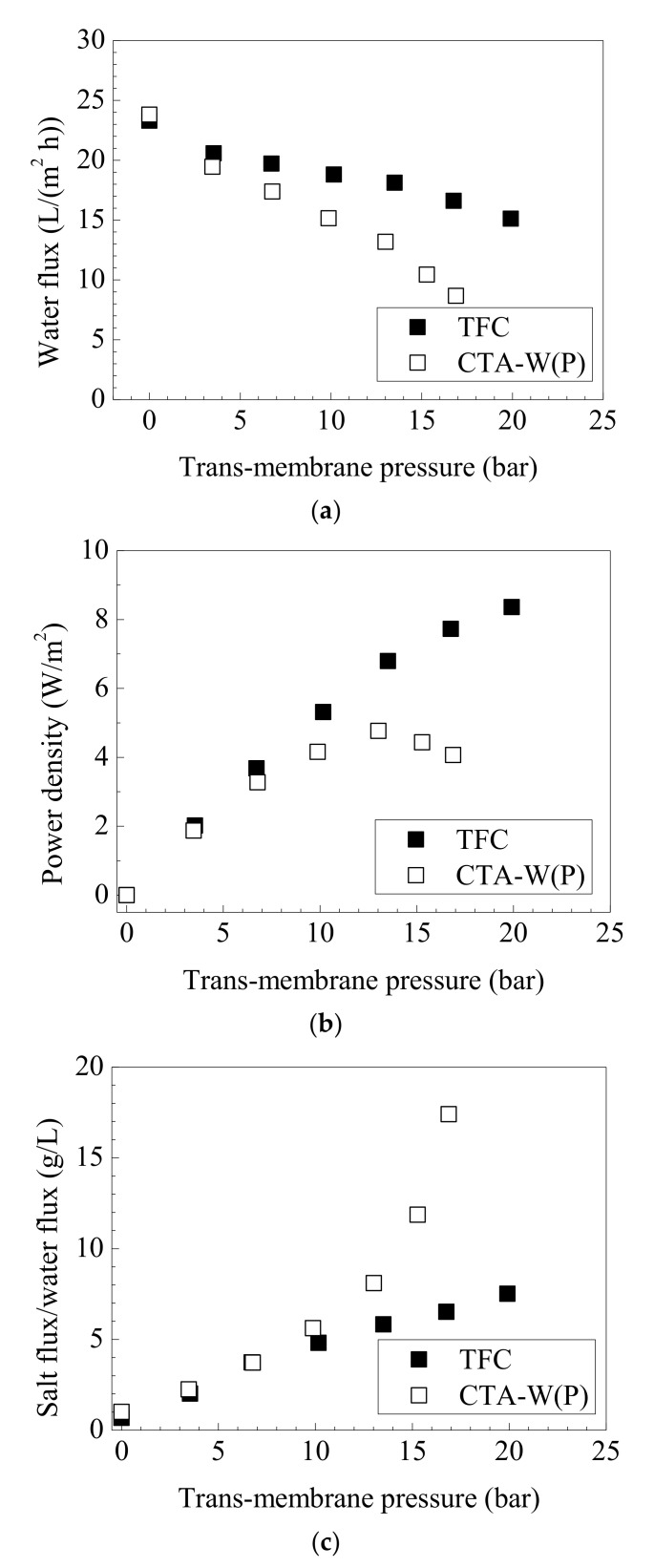
Pressure-retarded osmosis (PRO) performance of TFC and CTA-W(P) membranes: (**a**) water flux, (**b**) power density, and (**c**) salt flux/water flux ratio. Testing conditions: 10 mM NaCl as FS, 1 M NaCl as DS, and active-layer-facing-draw-solution (AL-DS) mode.

TFC and CTA-W(P) were tested in PRO process to estimate their efficiency of osmotic power production. Both membranes can withstand transmembrane pressure of 0–22 bar without burst. As Δ*P* gradually increased, water permeation through membrane was retarded. *J_v_* of TFC declines less than that of CTA-W(P) upon pressurization (Figure 6a). TFC achieved a peak power density of 8.4 W/m^2^ at Δ*P* of 20 bar (Figure 6b). The peak power density of CTA-W(P) was 4.8 W/m^2^ at Δ*P* of 13 bar. In PRO process, the theoretically maximum power density can be achieved at Δ*P* = 1/2 Δ*π*, which was 22 bar in the current testing condition (10 mM NaCl FS and 1 M NaCl DS). As in Figure 6c, *J_s_/J_v_* of CTA-W(P) sharply increased at Δ*P* > 10 bar. CTA-W(P) had relatively lower intrinsic salt rejection than TFC. Prior studies revealed that osmotic membranes are prone to rejection deterioration at high applied pressure due to the stretching of membranes [56]. CTA membrane with linear polymer network became loose after deformed and caused severe salt leakage. Therefore, the peak power density of CTA-W(P) was reached at a Δ*P* much lower than the theoretically optimal value. In contrast, TFC with superior salt rejection and a more rigid cross-linked polyamide rejection layer can minimize the influence of solute reverse diffusion and showed higher PRO performance.

## 4. Implications

**Figure 7:**
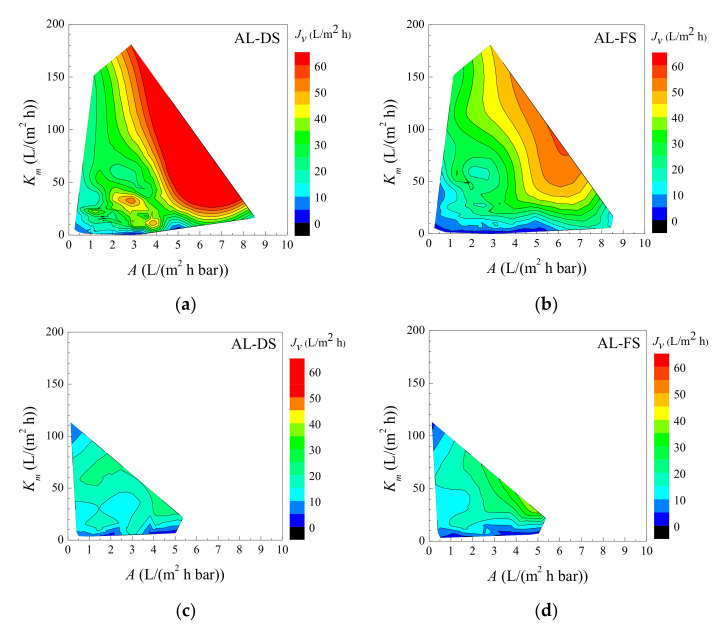
FO water flux of membranes with different water permeability (*A*) and mass diffusion coefficient (*K_m_*). The data was obtained from the experiments in the current work and the literature, and the membrane properties are presented in Table A1 and Table S1. Data in (**a**,**b**) were evaluated with FS of 0–10 mM NaCl and DS of 0.5–0.75 M NaCl. Data in (**c**,**d**) were evaluated with FS of 0.5–0.6 M NaCl and DS of 1.5–2 M NaCl.

The study of TFC and CTA-W(P) showed the non-linear dependence of OM water flux on membrane property. It was complicated with the trade-off of critical membrane properties, e.g., *A*, *B/A*, *S*, and mechanical strength, and increased the complexity of membrane optimization. FO membranes in the literature were investigated to study the comprehensive influence of membrane permeability and structure on *J_v_*. Experimental data of the membranes in the current work and the literature (Table A1, Tables S1 and S2) were plotted according to two potential applications: (1) dehydration of low salinity solutions using seawater as DS (Figure 7a,b), and (2) seawater desalination using high concentration DS (Figure 7c,d). As *J_v_* is positively related to both *A* and *K_m_*, *K_m_* was plotted instead of *S* for better illustration and understanding. The isolines present datapoints with the same *J_v_*, which were achieved by membranes with different *A* and *K_m_*. Higher *J_v_* were mostly exhibited by membranes with higher *A* and *K_m_*, if the solute reverse diffusion was low. Meanwhile, there would be a governing factor in a specific case, depending on the combination of *A* and *K_m_*. For example, when *K_m_* was smaller than 20 L/(m^2^ h) (i.e., S > 0.3 mm), the increase of *J_v_* with *K_m_* was more remarkable while increasing *A* had less effect on *J_v_* if the membranes had moderate *A* value. It means that improving membrane structure can significantly improve *J_v_*. On the other hand, for membranes with optimized structure (e.g., *K_m_* > 20 L/(m^2^ h)) but low water permeability (e.g., A < 2 L/(m^2^ h bar)), the increase of *J_v_* with *A* was more significant, suggesting that *A* was the governing factor on *J_v_*. The membrane property dominating OM performance also varied by testing condition. Taking CTA-W(P) (*A* = 1.06 L/(m^2^ h bar), *K_m_* = 7.9 L/(m^2^ h)) as an example, the constraint of its low water permeability on *J_v_* was crucial in the test with low concentration FS and AL-DS mode (Figure 7a). As this condition was frictional-resistance-dominated, improving the *A* value of CTA-W(P) can effectively increase *J_v_*. In contrast, the constraint of *K_m_* on *J_v_* became more important when seawater was used as FS (Figure 7c,d), i.e., *J_v_* varied more obviously with *K_m_* instead of *A* value, due to the dominant effect of ICP. Comparison of the above membranes showed that the dominant factor and mechanism for OM flux varied by membrane property and OM conditions.

**Figure 8:**
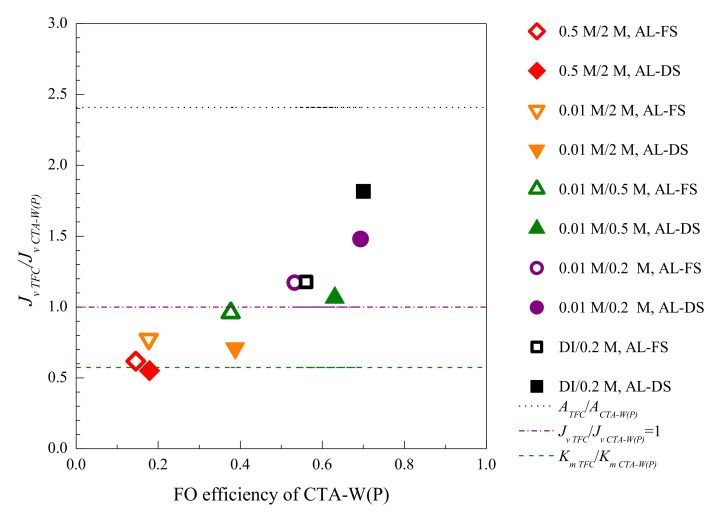
The FO efficiency of benchmark membrane (CTA-W(P)) and *J_v TFC_/J_v CTA-W(P)_* in different testing conditions. DI water, 0.01 M and 0.5 M NaCl were used as FS; 0.2 M, 0.5 M, and 2 M NaCl were used as DS, as in the legend.

A framework was proposed to illustrate the mechanisms dominating flux performance, and to guide the optimization of membranes for targeted OM applications. A few potential modes of OM applications have been studied in Section 3.3. The FO efficiency (*J_v_/J_v Theo_*) of CTA-W(P) varied with the FS, DS, and membrane orientation due to the comprehensive influence of concentration polarization, frictional resistance loss and solute reverse diffusion (Figure 8). With CTA-W(P) as a benchmark, pros and cons of TFC were assessed using this framework (Figure 8). When 10 mM NaCl FS and 0.5 M NaCl DS were used, moderate FO efficiency around 0.5 was showed by CTA-W(P). Both ICP and frictional resistance loss constrained the performance of CTA-W(P). TFC had comparable *J_v_* as CTA-W(P) under this condition, and using TFC as an alternative might not significantly enhance *J_v_*, but its excellent salt rejection would be beneficial to reduce the solute reverse diffusion. Higher FO efficiency is shown at the right side of the diagram. The corresponding OM conditions (e.g., tests with AL-DS mode, dilute FS and DS) had less ICP, and *J_v_* was likely to be governed by frictional resistance loss mechanism. *J_v TFC_/J_v CTA-W(P)_* sharply increased in the conditions with high FO efficiency. In an ideal case without ICP (FO efficiency = 1), *J_v_* will be linearly proportional to *A*. In Figure 8, *J_v TFC_/J_v CTA-W(P)_* tended to approximate *A_TFC_/A_CTA-W(P)_* at the right side of the diagram. For applications with less ICP, indicated by high FO efficiency, the influence of frictional resistance loss is critical. *J_v_* can be significantly increased by using membranes with higher *A* value (e.g., TFC).

Lower FO efficiency is shown at the left side of the diagram (Figure 8). Results of tests with high ICP potential, e.g., tests with AL-FS mode, concentrated FS or DS, are mostly in this area. TFC exhibited lower *J_v_* than *J_v CTA-W(P)_* here despite of the high *A_TFC_*, and *K_m_* was the governing factor. In harsh condition like FO desalination with seawater as FS and high concentration DS, Equations (4) and (5) can be derived as *J_v_* = *K_m_*ln(*π_draw_*/*π_feed_*) [49]. *J_v_* will be almost linearly proportional to *K_m_* as *π_draw_/π_feed_* is constant. The performance of TFC and CTA-W(P) with 0.5 M FS and 2 M DS agreed with this equation. *J_v TFC_/J_v CTA-W(P)_* tended to approximate *K_m TFC_/K_m CTA-W(P)_* in both AL-DS and AL-FS modes (Figure 8). Increasing *A* value of membrane would have minor effect on *J_v_* but might compromise the membrane selectivity. Therefore, in applications dominated by ICP, indicated by low FO efficiency of the benchmark membrane, optimization or selection of membrane should be targeted to have improved mass transfer coefficient. Moreover, active layer with higher salt rejection, and lower fouling propensity for systems with high saturation index, would be important to reduce the salt leakage and *J_s_*-induced ICP.

## 5. Conclusions

In this work, the influence of membrane permeability and structure on OM performance was studied by systematically characterizing and evaluating the OM performance of commercial osmotic membranes. The polyamide-based TFC membrane with superior water permeability and salt rejection had high water flux in the FO conditions with low ICP, as well as in PRO tests with high hydraulic pressure. In FO tests with more severe ICP, however, an integral asymmetric CTA membrane (CTA-W(P)) with optimized structure of support layer showed higher water flux, despite of its lower water permeability coefficient. In OM process, flux is highly non-linear with respect to membrane property. The trade-off of membrane properties imposes limitations on membrane optimization. This study established a framework based on the FO efficiency to identify the factors governing water flux in different conditions. High FO efficiency indicates the dominant effect of frictional resistance loss, and improvement of the membrane water permeability would be more effective to achieve higher water flux. On the other hand, low FO efficiency is usually exhibited in the ICP-dominated condition, and membranes with smaller structural parameter should be used to enhance OM performance.

## Figures and Tables

**Table 1:** The chemical property, structural property, and intrinsic separation property of membranes.

	Property	TFC	CTA-W(P)	CTA-W	CTA-NW
	Membrane material	PA/PSf/PET	CTA/PET	CTA/PET	CTA/PET
Structural property	Thickness (µm)	105 ± 4	71 ± 7 ^a^	61 ± 1 ^a^ (44.7 ± 14.1) ^b^	129 ± 6 (144 ± 24) ^b^
	Porosity (%)	66 ± 1	62 ± 1	50 ± 1 (46 ± 1) ^b^	55 ± 1 (50 ± 2) ^b^
	Structural parameter, *S* (mm)	1.14 ± 0.12	0.73 ± 0.31	0.82 ± 0.39 (1.00 ± 0.54) ^b^	1.77 ± 0.20 (1.38 ± 0.26) ^b^
Surface property	Roughness, *R_q_* (nm)	69 ± 15	5 ± 1	5 ± 1	33 ± 18
	Contact angle (°)	48 ± 3	63 ± 1	67 ± 2 (73 ± 2) ^b^	63 ± 4 (64 ± 2) ^b^
Mechanical property	Young’s modulus, *E* (MPa)				
	With tension in axial direction	158 ± 31	427 ± 34	384 ± 33	261 ± 70 (Isotropic)
	With tension in diagonal direction	38 ± 3	152 ± 13	264 ± 14	
	Tensile strain at break (%)				
	With tension in axial direction	67 ± 2	55 ± 4	65 ± 7	77 ± 11 (Isotropic)
	With tension in diagonal direction	151 ± 14	175 ± 16	150 ± 12	
	Tensile stress at break (MPa)				
	With tension in axial direction	54 ± 3	76 ± 9	92 ± 8	26 ± 5 (Isotropic)
	With tension in diagonal direction	32 ± 1	76 ± 2	95 ± 4	
Intrinsic separation property	Water permeability, *A* (L/(m^2^ h bar))	2.56 ± 0.08	1.06 ± 0.07	0.41 ± 0.01 (0.33 ± 0.04) ^b^	0.53 ± 0.03 (0.46 ± 0.07) ^b^
	NaCl rejection (%)	96.5 ± 0.9	88.1 ± 4.3	92.5 ± 0.3 (81.9) ^b,c^	93.9 ± 1.2 (92.4) ^b,c^
	NaCl permeability, *B_NaCl_* (L/(m^2^ h))	0.63 ± 0.21	0.67 ± 0.24	0.15 ± 0.01 (0.14 ± 0.03) ^b^	0.16 ± 0.04 (0.10 ± 0.01) ^b^
	*B_NaCl_/A* (bar)	0.25 ± 0.08	0.65 ± 0.27	0.38 ± 0.02 (0.47 ± 0.12) ^b^	0.31 ± 0.06 (0.22 ± 0.03) ^b^
	Na_2_SO_4_ rejection (%)	98.9 ± 0.2	96.4 ± 1.8	99.0 ± 0.04	98.8 ± 0.01
	Na_2_SO_4_ permeability, *B_Na_*_2*SO*4_ (L/(m^2^ h))	0.13 ± 0.02	0.17 ± 0.08	0.019 ± 0.0002	0.029 ± 0.001
	*B_Na_*_2*SO*4_*/A* (bar)	0.053 ± 0.008	0.17 ± 0.09	0.047 ± 0.001	0.055 ± 0.001

^a^ The cross-sections of CTA-W(P) and CTA-W were non-uniform due to the embedded mesh. Thickness at the thinnest parts were 52 ± 7 µm and 25 ± 1 µm for CTA-W(P) and CTA-W, respectively. ^b^ Values in parentheses were reported in previous study [24]. The values in current study were slightly different, likely due to variations of different membrane batches. ^c^ Salt rejection was tested at 3.75 bar, using 20 mM NaCl as feed in previous study [24].

## Supplementary Materials

The following are available online at https://www.mdpi.com/2077-0375/11/2/153/s1, S1. Setup for Membrane Permeability Measurement, S2. Surface Morphology of TFC Membrane after Scaling Test, S3. Property and Performance of Lab-Scale FO Membranes in the Literature.

## Appendix A. Structure of CTA-W and CTA-NW Membranes

Cross-sections of CTA-W and CTA-NW are shown in Figure A1. CTA-W had a similar structure as CTA-W(P), consisting of a CTA rejection layer and a mesh embedded in the CTA layer. Compared to CTA-W(P), the CTA layer of CTA-W was thinner and denser without macrovoid, which resulted in a slightly larger *S* value of 0.82 mm. CTA-NW comprised a CTA layer and a nonwoven fabric (thickness of ~80 μm) supported on the bottom. Finger-like pore structure was shown in the CTA layer. Due to the thick and dense nonwoven fabric support, CTA-NW had a smaller porosity of 55% and thicker cross-section (129 mm) than CTA-W(P), as well as had a large *S* value of 1.77 mm.

## Appendix B. Tensile Strength Tests

Mechanical property of the mesh-reinforced TFC, CTA-W(P), and CTA-W membranes was measured with tension in the axial direction along filaments, and the diagonal direction of mesh opening, as shown in Figure A2.

## Appendix C. Property and Performance of Commercial FO Membranes

The separation property and FO performance of membranes in this study was compared with other commercial FO membranes reported in the literature. TFC membranes generally showed higher water permeability and selectivity than integral asymmetric membranes (Table A1). With support layer tailored to have small structural parameter, the TFC membranes achieved high water flux in OM process.

**Table A1:** Property and FO performance of commercial membranes.

	Membrane ID	Manufacturer	Membrane Structure	Membrane Material	Membrane Property				FO Performance and Testing Conditions						Reference
					*A* (L/(m^2^ h bar))	*B_NaCl_* (L/(m^2^ h))	*B_NaCl_/A* (bar)	*S* (mm)	*J_v_* (L/(m^2^ h))	*J_s_* (g/(m^2^ h))	*J_s_/J_v_* (g/L)	Membrane Orientation	Feed Solution	Draw Solution	
1	TFC	HTI	TFC flat-sheet membrane with mesh	PA/PSf/PET	2.56 ± 0.08	0.63 ± 0.21	0.25 ± 0.08	1.14 ± 0.12	15.82 ± 2.01	4.54 ± 2.44	0.28 ± 0.13	AL-DS	10 mM NaCl	0.5 M NaCl	Current work
									8.51 ± 0.25	2.74 ± 0.99	0.32 ± 0.11	AL-FS	10 mM NaCl	0.5 M NaCl	
									8.07 ± 1.81	N.A.	N.A.	AL-DS	0.5 M NaCl	2 M NaCl	
									7.35 ± 0.83	N.A.	N.A.	AL-FS	0.5 M NaCl	2 M NaCl	
2	CTA-W(P)	HTI	Asymmetric flat-sheet membrane with mesh	CTA/PET	1.06 ± 0.07	0.67 ± 0.24	0.65 ± 0.27	0.73 ± 0.31	14.85 ± 0.34	12.80 ± 1.88	0.86 ± 0.14	AL-DS	10 mM NaCl	0.5 M NaCl	
									8.88 ± 0.41	7.05 ± 0.22	0.80 ± 0.06	AL-FS	10 mM NaCl	0.5 M NaCl	
									14.65 ± 0.64	N.A.	N.A.	AL-DS	0.5 M NaCl	2 M NaCl	
									11.90 ± 0.69	N.A.	N.A.	AL-FS	0.5 M NaCl	2 M NaCl	
3	CTA-W	HTI	Asymmetric flat-sheet membrane with mesh	CTA/PET	0.41 ± 0.01	0.15 ± 0.01	0.38 ± 0.02	0.82 ± 0.39	7.07 ± 0.32	3.78 ± 0.62	0.54 ± 0.10	AL-DS	10 mM NaCl	0.5 M NaCl	
									5.16 ± 0.17	1.94 ± 1.24	0.37 ± 0.24	AL-FS	10 mM NaCl	0.5 M NaCl	
									9.71 ± 0.85	N.A.	N.A.	AL-DS	0.5 M NaCl	2 M NaCl	
									8.32 ± 0.05	N.A.	N.A.	AL-FS	0.5 M NaCl	2 M NaCl	
4	CTA-NW	HTI	Asymmetric flat-sheet membrane with nonwoven fabric	CTA/PET	0.53 ± 0.03	0.16 ± 0.04	0.31 ± 0.06	1.77 ± 0.20	7.03 ± 0.37	2.37 ± 1	0.35 ± 0.01	AL-DS	10 mM NaCl	0.5 M NaCl	
									3.54 ± 0.21	1.03 ± 0.27	0.29 ± 0.09	AL-FS	10 mM NaCl	0.5 M NaCl	
									4.41 ± 0.48	N.A.	N.A.	AL-DS	0.5 M NaCl	2 M NaCl	
									4.14 ± 0.23	N.A.	N.A.	AL-FS	0.5 M NaCl	2 M NaCl	
5	Oasys	Oasys Water	TFC flat-sheet membrane with fabric	PA/PSf/PET	4.25 ± 0.04	0.38 ± 0.11	0.089 ^a^	0.483 ± 0.079	~32 ^b^	~7 ^b^	0.22 ^a^	AL-DS	DI water	~0.5 M NaCl	[28]
									~15 ^b^	~2 ^b^	0.13 ^a^	AL-FS	DI water	~0.5 M NaCl	
6	Porifera	Porifera	TFC flat-sheet membrane with mesh	PA-based	2.2	0.50	0.23 ^a^	0.344	15.6 ± 0.5	10.30 ^a^	0.66 ± 0.02	AL-FS	DI water	0.5M NaCl	[31]
7	WJ-FO	Woongjin Chemical	TFC flat-sheet membrane	PA-based	2.7 ± 1.12	0.83 ± 0.47	0.322	0.55 ± 0.21	~11 ^b^	16.5 ^a^	1.5	AL-FS	DI water	0.5 M NaCl	[30]
8	TCK-N	Toray Chemical Korea	TFC flat-sheet membrane with nonwoven fabric	PA/PSf/PET	6.601	1.19	0.18 ^a^	0.461	~56 ^b^	~34 ^b^	0.61 ^a^	AL-DS	DI water	1 M NaCl	[29]
									~28 ^b^	~15 ^b^	0.54 ^a^	AL-FS	DI water	1 M NaCl	
9	TCK-W	Toray Chemical Korea	TFC flat-sheet membrane with mesh	PA/PSf/PET	5.362	0.95	0.18 ^a^	0.266	~64 ^b^	~25 ^b^	0.39 ^a^	AL-DS	DI water	1 M NaCl	
									~38 ^b^	~12 ^b^	0.32 ^a^	AL-FS	DI water	1 M NaCl	
10	Aquaporin Inside	Aquaporin A/S, Denmark	TFC flat-sheet membrane	Aquaporin-PA-based	0.52 ± 0.06	0.09	0.17 ^a^	0.825	~8 ^b^	~3 ^b^	~0.4 ^b^	AL-DS	DI water	0.5 M NaCl	[32]
									~5 ^b^	~1.5 ^b^	~0.32 ^b^	AL-FS	DI water	0.5 M NaCl	
11	HF-A	Toyobo	Asymmetric hollow fiber membrane	CTA	0.266	0.08	0.30	1.024	~6 ^b^	N.A.	N.A.	AL-DS	DI water	0.6 M NaCl	[25]
									~3 ^b^	N.A.	N.A.	AL-FS	DI water	0.6 M NaCl	
12	HF-B	Toyobo	Asymmetric hollow fiber membrane	CTA	0.291	0.02	0.069	0.724	~6 ^b^	N.A.	N.A.	AL-DS	DI water	0.6 M NaCl	
									~4 ^b^	N.A.	N.A.	AL-FS	DI water	0.6 M NaCl	
13	HF-C	Toyobo	Asymmetric hollow fiber membrane	CTA	0.545	0.04	0.073	0.639	~10 ^b^	N.A.	N.A.	AL-DS	DI water	0.6 M NaCl	
									~6 ^b^	N.A.	N.A.	AL-FS	DI water	0.6 M NaCl	

^a^ The value was calculated based on the data provided in references. ^b^ Data were obtained from the figures in the references.

## Appendix D. FO Performance of Membranes

FO performance of the membranes were tested with DS of 0.5 M and 2 M NaCl, and FS of 10 mM NaCl (Figure A3). Comparable *J_v_* were shown by TFC and CTA-W(P) with 0.5 M NaCl DS. TFC had remarkably lower *J_s_/J_v_* due to the superior salt rejection of polyamide rejection layer. Nevertheless, TFC had lower *J_v_* than CTA-W(P) when 2 M DS was used. Due to the constraint of low *A* values, CTA-W and CTA-NW had low *J_v_* in the tests. CTA-NW with large *S* value encountered more severe ICP and showed the lowest *J_v_* of all the membranes.
