# Supplementary material for: Insights into the Influence of Membrane Permeability and Structure on Osmotically-Driven Membrane Processes
Source: Membranes (Basel). 2021 Feb 22;11(2):153. doi: 10.3390/membranes11020153 (PMC7926744; doi:10.3390/membranes11020153)
Supplement: Supplementary file 1 [file membranes-11-00153-s001.pdf]

membranes

Supporting Information

Insights into the Influence of Membrane Permeability and Structure on Osmotically-Driven Membrane Processes

Jing Wei [1,2,3], Qianhong She [3,4,*] and Xin Liu [3]

[1] School of the Environment and Safety Engineering, Jiangsu University, 301 Xuefu Road, Zhenjiang 212013, Jiangsu, China; weijing@ujs.edu.cn
[2] Institute of Environmental Health and Ecological Security, Jiangsu University, 301 Xuefu Road, Zhenjiang 212013, Jiangsu, China
[3] Singapore Membrane Technology Centre, Nanyang Technological University, 1 Cleantech Loop, Singapore 637141, Singapore; liux5@sustc.edu.cn
[4] School of Civil and Environmental Engineering, Nanyang Technological University, 50 Nanyang Avenue, Singapore 639798, Singapore
* Correspondence: qhshe@ntu.edu.sg

S1. Setup for Membrane Permeability Measurement

A cross-flow filtration setup was used to determine the membrane permeability in reverse osmosis (RO) mode (Figure S1). Membrane sample was installed in the filtration cell with effective membrane area of 42 cm^2. Feed was pumped into the cell and flowed on the active layer of membrane. The concentrate and permeate were returned to the feed tank, and circulated in the system. Sample of permeate was taken to measure the water permeability coefficient, salt permeability coefficient, and salt rejection of membrane.

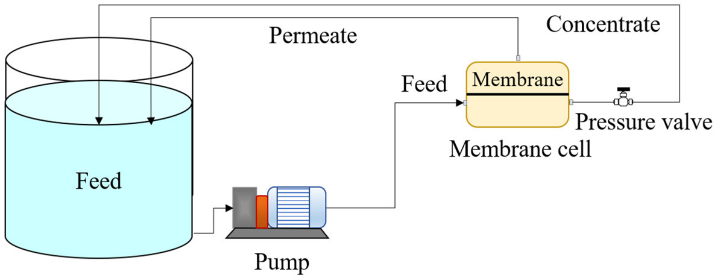

Figure S1. Schematic diagram of cross-flow reverse osmosis (RO) setup.

S2. Surface Morphology of TFC Membrane after Scaling Test

The surface of thin film composite membrane (TFC) was observed using field-emission scanning electron microscopy (FESEM) after scaling test. Damage of the active layer can be seen in Figure S2. TFC was susceptible to scaling because of the surface roughness and chemical property. Growth of gypsum crystals at the confined zone near spacer resulted in damage of the ultrathin active layer.

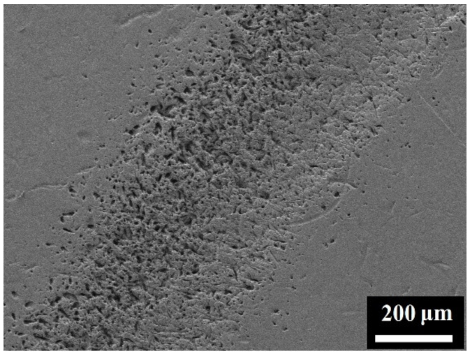

Figure S2. Field-emission scanning electron microscopy (FESEM) micrograph of thin film composite membrane (TFC) active layer (at 100x) after scaling test.

S3. Property and Performance of Lab-Scale FO Membranes in the Literature

Lab-scale forward osmosis (FO) membranes in the literature were investigated. Their water permeability, salt permeability and structural parameter are listed in Table S1. The abbreviations of membrane materials are shown in Table S2. To study the influence of membrane permeability and structure on water flux, FO performance of these membranes was summarized in Table S1.

Table S1. Property and performance of lab-scale FO membranes in the literature.

	Membrane ID	Membrane structure	Membrane material	Membrane property				FO performance and testing conditions						Reference
				A (L/(m^2 h bar))	B_{NaCl} (L/(m^2 h))	B_{NaCl}/A (bar)	S (mm)	J_v (L/(m^2 h))	J_s (g/(m^2 h))	J_s/J_v (g/L)	Membrane orientation	Feed solution	Draw solution	
1	TFN0.008	TFC flat-sheet membrane	PA-GO/PSf	3.9 ± 0.2	1.1 ± 0.2	0.28 [b]	0.119 ± 0.0004	34.3 ± 0.1	1.1 ± 0.2	0.03	AL-FS	DI water	2 M NaCl	[1]
2	UiO-66-2/GO-1	TFC flat-sheet membrane	UiO-66-GO/nylon	73.20	0.11	0.0015	0.00103	29.16 ± 0.28	12.86 ± 0.82	0.44 [b]	AL-FS	DI water	2 M NaCl	[2]
3	TFC-30	TFC flat-sheet membrane	PA-AEPPS/PSf	4.81 ± 0.03	0.19 ± 0.01	0.039	0.747	27	12	0.45	AL-DS	DI water	1 M NaCl	[3]
								16	6	0.35	AL-FS	DI water	1 M NaCl	
4	DPE-TFC	TFC flat-sheet membrane	PA/PDA-PE	6.67 ± 0.15	0.68 ± 0.02	0.103 ± 0.004	0.168 ± 0.004	64.8	18.1 [b]	0.28	AL-DS	DI water	1 M NaCl	[4]
								53.0	14.8 [b]	0.28	AL-FS	DI water	1 M NaCl	
5	TFC$_{TiO_2}$/GO	TFC flat-sheet membrane	PA/PSf-TiO_2-GO	0.58 ± 0.01	0.052 ± 0.012 [b]	0.089 [b]	0.20 ± 0.01	21.0	~3 [a]	~0.14 [a]	AL-DS	DI water	0.5 M NaCl	[5]
								12.3	~1 [a]	~0.08 [a]	AL-FS	DI water	0.5 M NaCl	
6	TFN-MMGO/Fe_3O_4-100	TFC flat-sheet membrane	PA-GO-Fe_3O_4/PES	2.69	0.26	0.10	N.A.	~62 [a]	~10 [a]	~0.16 [a]	AL-DS	DI water	1 M NaCl	[6]
								~55 [a]	~10 [a]	~0.18 [a]	AL-FS	DI water	1 M NaCl	
7	TFN0.05	TFC flat-sheet membrane	PA-g-C_3N_4/PSf	2.17 ± 0.18	0.38 [b]	0.179 ± 0.04	0.37 ± 0.09	18.88 ± 0.35	2.74 ± 0.15	0.15 [b]	AL-FS	DI water	2 M NaCl	[7]
8	PE-TFC	TFC flat-sheet membrane	PA/PE	3.15 ± 0.17	0.48 ± 0.19	0.133 ± 0.058	0.161	~32 [a]	~15 [a]	~0.5 [a]	AL-DS	DI water	0.5 M NaCl	[8]
								~28 [a]	~14 [a]	~0.5 [a]	AL-FS	DI water	0.5 M NaCl	

	Membrane ID	Membrane structure	Membrane material	Membrane property				FO performance and testing conditions						Reference
				A (L/(m^2 h bar))	B_{NaCl} (L/(m^2 h))	B_{NaCl}/A (bar)	S (mm)	J_v (L/(m^2 h))	J_s (g/(m^2 h))	J_s/J_v (g/L)	Membrane orientation	Feed solution	Draw solution	
9	5wt%IER-Na	TFC flat-sheet membrane	PA/PSf-(IER-Na)	3.72	0.1078	0.03 [b]	0.742	43.8	~6 [a]	0.14	AL-DS	DI water	1.5 M NaCl	[9]
								~25 [a]	~5 [a]	~0.2 [a]	AL-FS	DI water	1.5 M NaCl	
10	TFN-ZSCSNP-1	TFC flat-sheet membrane	PA/PES-ZSCSNPs	3.47 ± 0.09	4.01 ± 0.08	1.1556 ± 0.0008	0.297 ± 0.012	25.93	~10 [a]	0.39 [b]	AL-FS	DI water	0.5 M NaCl	[10]
11	10 wt% PVDF (DMAc/ Water = 50:50)	TFC flat-sheet membrane with dual-layer nanofiber substrate	PA/PVDF/ PET-PVA	2.02 ± 0.20	1.09 ± 0.09	0.54 [b]	0.243 ± 0.0421	23.57 ± 1.08	~15 [a]	0.64 [b]	AL-DS	DI water	0.5 M NaCl	[11]
								~20 [a]	~13 [a]	0.65 [b]	AL-FS	DI water	0.5 M NaCl	
12	TFC-SUB 2	TFC flat-sheet membrane with mesh	PA/PVC-LDH/polyester	3.61 ± 0.019	0.1816 ± 0.033	0.05 [b]	0.303	50.89 ± 1.13	13.284 ± 0.67	0.26 [b]	AL-DS	DI water	1 M NaCl	[12]
								37.46 ± 0.85	3.57 ± 0.2	0.10 [b]	AL-FS	DI water	1 M NaCl	
13	MT-3	TFC flat-sheet membrane	PA/PVDF-PFSA	2.97 ± 0.06	0.39 ± 0.13	0.1284 ± 0.0422	0.33462 ± 0.00350	18.8	~5 [a]	0.27 [b]	AL-FS	DI water	0.5 M NaCl	[13]
14	TFN-0.04	TFC flat-sheet membrane	PA-MOF/PES	~5 [c]	~0.6 [c]	0.12 [c]	0.238	~42 [a]	N.A.	N.A.	AL-DS	DI water	0.5 M NaCl	[14]
								~30 [a]	~4 [a]	~0.12 [a]	AL-FS	DI water	0.5 M NaCl	
15	VOPS-TFC-1	TFC flat-sheet membrane	PA/PVDF	4.71 ± 0.22	N.A.	N.A.	0.0991 ± 0.0037	~65 [a]	~5 [a]	0.08 [b]	AL-DS	DI water	0.5 M NaCl	[15]
								~45 [a]	~5 [a]	0.11 [b]	AL-FS	DI water	0.5 M NaCl	

	Membrane ID	Membrane structure	Membrane material	Membrane property				FO performance and testing conditions						Reference
				A (L/(m² h bar))	B_{NaCl} (L/(m² h))	B_{NaCl}/A (bar)	S (mm)	J_v (L/(m² h))	J_s (g/(m² h))	J_s/J_v (g/L)	Membrane orientation	Feed solution	Draw solution	
16	SPSFcoTFC	TFC flat-sheet membrane with dual-layer substrate	PA/PSf-SPEEK/polyether imide	2.16 ± 0.13	0.16 ± 0.05	0.10 [b]	0.191 ± 0.047	~20 [a]	N.A.	N.A.	AL-DS	DI water	0.5 M NaCl	[16]
								22.4 ± 2.1	3.58 [b]	0.16 ± 0.05	AL-FS	DI water	0.5 M NaCl	
17	TFN-U2	TFC flat-sheet membrane	PA-MOF/PSf	3.33 ± 0.48	0.33 ± 0.05	0.10 ± 0.005	0.532	29.4	N.A.	N.A.	AL-DS	DI water	0.5 M NaCl	[17]
								18.4	N.A.	N.A.	AL-FS	DI water	0.5 M NaCl	
18	AQP-TFC-HF-PEI	TFC flat-sheet membrane	PA-aquaporin/polyether imide	7.6	~0.5 [a]	0.07 [b]	0.172 ± 0.006	64.7	8.3	0.13	AL-DS	DI water	0.5 M NaCl	[18]
								35.4	3.6	0.10	AL-FS	DI water	0.5 M NaCl	
19	TFC-PSf$_a$GO	TFC flat-sheet membrane with dual-layer substrate	PA/PSf-GO/PSf-GO	1.46	0.25	0.17	0.130	61.5	~12 [a]	0.18	AL-DS	DI water	1 M NaCl	[19]
								33.8	~7 [a]	0.19	AL-FS	DI water	1 M NaCl	
20	PS$_{0.5}$-TFN$_{0.05}$	TFC flat-sheet membrane with mesh	PA/PSf-Al_2O_3/PET	8.43	1.66	0.20	1.028	~15 [a]	N.A.	N.A.	AL-FS	DI water	0.5 M NaCl	[20]
21	PA/PAN-eTFC	TFC flat-sheet membrane with nanofiber substrate	PA/PAN	1.47 [c]	0.278 [c]	0.19 [b]	0.168	~18 [a]	~4 [a]	0.22 [b]	AL-FS	DI water	0.5 M NaCl	[21]
22	PK(35/200)	TFC flat-sheet membrane	PA/PK	2.79 ± 0.05	0.54 ± 0.02	0.19 [b]	0.176 ± 0.004	30.3 ± 0.5	4.56 ± 0.70	0.15 [b]	AL-FS	DI water	0.6 M NaCl	[22]

	Membrane ID	Membrane structure	Membrane material	Membrane property				FO performance and testing conditions						Reference
				A (L/(m^2 h bar))	B_{NaCl} (L/(m^2 h))	B_{NaCl}/A (bar)	*S* (mm)	J_v (L/(m^2 h))	J_s (g/(m^2 h))	J_s/J_v (g/L)	Membrane orientation	Feed solution	Draw solution	
23	TFC-1.5	TFC flat-sheet membrane	PA/PAN	1.439 ± 0.006	0.197 ± 0.002	0.137 ± 0.001	0.298	~23 [a]	~16 [a]	0.70 [b]	AL-DS	DI water	0.5 M NaCl	[23]
								16.7	10	0.60 [b]	AL-FS	DI water	0.5 M NaCl	
24	TFC-25.0	TFC flat-sheet membrane	PA/PSf	1.57 ± 0.11	0.32 ± 0.10	0.20 [b]	0.397	~35 [a]	~5 [a]	0.14 [b]	AL-DS	DI water	0.5 M NaCl	[24]
							0.186	~20 [a]	~5 [a]	0.25 [b]	AL-FS	DI water	0.5 M NaCl	
25	PA/PVDF/CA (8%)	TFC flat-sheet membrane with mesh-embedded dual-layer substrate	PA/PVDF/CA/polyester	1.2 ± 0.2	0.40 ± 0.03	0.33 [b]	0.391	~25 [a]	~5 [a]	0.20 [b]	AL-DS	DI water	0.5 M NaCl	[25]
								~12 [a]	~4 [a]	0.33 [b]	AL-FS	DI water	0.5 M NaCl	
26	Pa-Si15	TFC flat-sheet membrane with nonwoven-fabric-supported nanofiber substrate	PA/PAN-silica nanoparticles (NPs)/PET	2.54 ± 0.86	1.66 ± 0.47	0.65 [b]	0.065 ± 0.005	~82 [a]	~12 [a]	~0.15 [a]	AL-DS	DI water	1 M NaCl	[26]
								55.98	7.98	~0.15 [a]	AL-FS	DI water	1 M NaCl	
27	TFC3 -20 ºC TMC	TFC flat-sheet membrane	PA/PES	5.78	4.96	0.86 [b]	0.436	~21 [a]	N.A.	N.A.	AL-FS	DI water	0.5 M NaCl	[27]
28	nTFC0.15	TFC flat-sheet membrane	PA-CNTs-PDA/PSf	6.5	7.0 [d]	1.08 [b,d]	1.669	~13 [a]	~7 [a]	0.54 [b]	AL-FS	DI water	2 M $MgCl_2$	[28]

	Membrane ID	Membrane structure	Membrane material	Membrane property				FO performance and testing conditions						Reference
				A (L/(m^2 h bar))	B_{NaCl} (L/(m^2 h))	B_{NaCl}/A (bar)	S (mm)	J_v (L/(m^2 h))	J_s (g/(m^2 h))	J_s/J_v (g/L)	Membrane orientation	Feed solution	Draw solution	
29	TFNC-2	TFC flat-sheet membrane	PA/PSf-LDH NPs	0.61 [c]	0.27 [c]	0.45	0.148	34.6	12.7	0.37 [b]	AL-DS	DI water	1 M NaCl	[29]
								18.1	8.1	0.45 [b]	AL-FS	DI water	1 M NaCl	
30	#1 sPPSU-TFC	TFC flat-sheet membrane with mesh	PA/sPPSU /PET	3.7 ± 0.38	0.228 ± 0.012	0.06 [b]	0.256	~42 [a]	10.5 [b]	~0.25 [a]	AL-DS	DI water	~0.6 M NaCl	[30]
								~26 [a]	6.5 [b]	~0.25 [a]	AL-FS	DI water	~0.6 M NaCl	
31	Modified -TFC	TFC flat-sheet membrane with nonwoven-fabric-supported nanofiber substrate	PA/PVDF-nylon 6,6/PET	1.28 ± 0.36	0.25 ± 0.11 [c]	0.20 [b]	0.193 ± 0.022	~20 [a]	~7 [a]	0.35 [b]	AL-DS	DI water	0.5 M NaCl	[31]
								~15 [a]	~2 [a]	0.13 [b]	AL-FS	DI water	0.5 M NaCl	
32	TFC-flat	TFC flat-sheet membrane	PA/PSf	1.58 ± 0.04	0.17 ± 0.09	0.11 [b]	0.226 ± 0.057	18.1 ± 1.5	N.A.	N.A.	AL-FS	DI water	0.5 M NaCl	[32]
33	PES/PAA 5/$CaCO_3$	TFC flat-sheet membrane	PA/PES-PAA-$CaCO_3$	~0.75	N.A.	N.A.	0.0357	62	21.6	~0.35 [a]	AL-DS	DI water	2 M NaCl	[33]
								52	16.8	~0.33 [a]	AL-FS	DI water	2 M NaCl	
34	TFN 0.5	TFC flat-sheet membrane	PA/PSf-HNT	2.00	0.34	0.1680	0.37 ± 0.05	26.91	8.50	0.32 [b]	AL-DS	10 mM NaCl	0.5 M NaCl	[34]
								14.88	5.95	0.40 [b]	AL-FS	10 mM NaCl	0.5 M NaCl	
35	Dual	TFC flat-sheet membrane	PA/CAP/PA	0.98 ± 0.01	0.09 ± 0.01 [c]	0.09 [b]	0.052 ± 0.008	40.4 ± 0.7	5.7 ± 0.4	0.14 [b]	AL-DS	DI water	1 M NaCl	[35]

	Membrane ID	Membrane structure	Membrane material	Membrane property				FO performance and testing conditions						Reference
				A (L/(m^2 h bar))	B_{NaCl} (L/(m^2 h))	B_{NaCl}/A (bar)	S (mm)	J_v (L/(m^2 h))	J_s (g/(m^2 h))	J_s/J_v (g/L)	Membrane orientation	Feed solution	Draw solution	
		with double-skinned substrate						34.5 ± 1.7	3.5 ± 0.6	0.10 [b]	AL-FS	DI water	1 M NaCl	
36	GOT-0.25	TFC flat-sheet membrane	PA/PSf-GO	1.76	0.19	0.11	0.191	40.50	~6.5 [a]	0.16 [b]	AL-DS	DI water	0.5 M NaCl	[36]
								19.77	~3.5 [a]	0.18 [b]	AL-FS	DI water	0.5 M NaCl	
37	PMM_{C300}	Layer-by-layer (LbL) flat-sheet membrane	PAH-PSS/PAN-MOF	~8 [a]	N.A.	N.A.	0.19 ± 0.02	107.4	~0.17 [a]	0.002 [b]	AL-DS	DI water	0.5 M $MgCl_2$	[37]
								~38 [a]	~0.3 [a]	0.01 [b]	AL-FS	DI water	0.5 M $MgCl_2$	
38	Hydrogel/GO	Composite flat-sheet membrane	Hydrogel/PES-GO	1.52 ± 0.12	N.A.	N.A.	0.197 ± 0.021	16.05 ± 1.40	1.27 ± 0.44	0.08 [b]	AL-FS	DI water	0.5 M Na_2SO_4	[38]
39	mLbL-10 TFC	TFC flat-sheet membrane with nonwoven fabric	PA/PEI-PAA/PAN/PET	2.72 ± 0.06	1.07 ± 0.10	0.395 ± 0.045	0.35	32.9	3.77	0.11	AL-DS	DI water	0.5 M NaCl	[39]
								24.6	2.36	0.10	AL-FS	DI water	0.5 M NaCl	
40	PSf_{co}-TFC	TFC flat-sheet membrane	PA/PSf	1.65 ± 0.06	0.12 ± 0.05	0.07 [b]	0.167 ± 0.016	33.1 ± 1.4	2.58 [b]	0.078	AL-DS	DI water	0.5 M NaCl	[40]
								20.1 ± 0.9	2.01 [b]	~0.1 [a]	AL-FS	DI water	0.5 M NaCl	
41	CN/rGO-M-0.5	TFC flat-sheet membrane	PA/PSf-CN/rGO	1.59 [c]	0.329 [c]	0.21	0.163	~21 [a]	~5 [a]	0.24 [b]	AL-FS	DI water	0.5 M NaCl	[41]
42	TFC-D4	TFC flat-sheet membrane	PA/PSf/PET	1.82 ± 0.11	0.28 ± 0.10	0.15	0.195 ± 0.039	60.3	17.6	0.29 [b]	AL-DS	DI water	1 M NaCl	[42]

	Membrane ID	Membrane structure	Membrane material	Membrane property				FO performance and testing conditions						Reference
				A (L/(m^2 h bar))	B_{NaCl} (L/(m^2 h))	B_{NaCl}/A (bar)	S (mm)	J_v (L/(m^2 h))	J_s (g/(m^2 h))	J_s/J_v (g/L)	Membrane orientation	Feed solution	Draw solution	
		with mesh-reinforced dual-layer substrate						31.1	8.5	0.27 [b]	AL-FS	DI water	1 M NaCl	
43	TFC/PSF9	TFC flat-sheet membrane with mesh	PA/PSf/polyester	0.91 ± 0.10 [c]	0.25 ± 0.04 [c]	0.27 [b]	0.314 ± 0.029	49.4	~6 [a]	0.12 [b]	AL-DS	DI water	1 M NaCl	[43]
								17.1	~6 [a]	0.35 [b]	AL-FS	DI water	1 M NaCl	
44	TFC-O-II	TFC flat-sheet membrane	PA/CAP	2.85 [c]	0.345 [c]	0.12 [b]	0.0319	~60 [a]	~7 [a]	0.12 [b]	AL-DS	DI water	0.5 M NaCl	[44]
								~45 [a]	~6 [a]	0.13 [b]	AL-FS	DI water	0.5 M NaCl	
45	SPPO/PSf (50:50) – 45 s	TFC flat-sheet membrane	PA/PSf-SPPO	3.55	0.74	0.21 [b]	0.381 ± 0.098	~35 [a]	~5 [a]	0.14 [b]	AL-DS	DI water	0.5 M NaCl	[45]
							0.293 ± 0.022	~22 [a]	~5 [a]	0.23 [b]	AL-FS	DI water	0.5 M NaCl	
46	NC-PVA/PA	TFC flat-sheet membrane with nonwoven-fabric-reinforced nanofiber	PA/PVA/PET	1.69	0.24	0.14 [b]	0.066 ± 0.0079	27.24	N.A.	N.A.	AL-FS	DI water	0.5 M NaCl	[46]
47	TFN	TFC flat-sheet membrane	PA/PSf-TiO_2 NPs	1.96	0.38	0.1955	0.42	31.1	6.43	0.22	AL-DS	10 mM NaCl	0.5 M NaCl	[47]
								17.1	3.97	0.16	AL-FS	10 mM NaCl	0.5 M NaCl	

	Membrane ID	Membrane structure	Membrane material	Membrane property				FO performance and testing conditions						Reference
				A (L/(m^2 h bar))	B_{NaCl} (L/(m^2 h))	B_{NaCl}/A (bar)	S (mm)	J_v (L/(m^2 h))	J_s (g/(m^2 h))	J_s/J_v (g/L)	Membrane orientation	Feed solution	Draw solution	
48	TFI-$M_{1.1}$	Thin film inorganic flat-sheet membrane	Silica xerogels/stainless steel mech	1.15	0.648	0.56 [b]	0.038	~25 [a]	4.25 [b]	~0.17 [a]	AL-DS	DI water	0.5 M NaCl	[48]
								~25 [a]	4.25 [b]	~0.17 [a]	AL-FS	DI water	0.5 M NaCl	
49	TFN 0.1	TFC flat-sheet membrane with nonwoven fabric	PA-F-MWCNTs/PSf/PET	4.47 ± 0.24	0.170 ± 0.025	0.042 ± 0.03	0.41 ± 0.1	95	~5 [a]	0.05 [b]	AL-DS	10 mM NaCl	2 M NaCl	[49]
								40	~3 [a]	0.08 [b]	AL-FS	10 mM NaCl	2 M NaCl	
50	sPPSU-2,5	TFC flat-sheet membrane	PA/sPPSU	3.23	1.05	0.33 [b]	0.652	~32 [a]	~5 [a]	0.16 [b]	AL-DS	DI water	0.5 M NaCl	[50]
								~30 [a]	~5 [a]	0.17 [b]	AL-FS	DI water	0.5 M NaCl	
51	TFC-R	TFC flat-sheet membrane	PA/PSf	3.46 ± 0.34	0.40 ± 0.06	0.11 ± 0.01	0.87 ± 0.18	~25 [a]	N.A.	N.A.	AL-DS	10 mM NaCl	0.75 M NaCl	[51]
								~13 [a]	N.A.	N.A.	AL-FS	10 mM NaCl	0.75 M NaCl	
52	-$N(CH_3)_3^+$	TFC flat-sheet membrane with nonwoven fabric	PA-silica NPs/PSf/PET	~2.5 [a]	~1.7 [a]	0.68 [b]	~0.5 [a]	19.5	N.A.	N.A.	AL-FS	DI water	1 M NaCl	[52]
53	TFN0.1	TFC flat-sheet membrane	PA- NaY Zeolite NPs/PSf	2.57	1.57	0.611	0.782 ± 0.160	~22 [a]	N.A.	N.A.	AL-DS	DI water	0.5 M NaCl	[53]
								~12 [a]	N.A.	N.A.	AL-FS	DI water	0.5 M NaCl	
54	TMC 0.05	TFC flat-sheet membrane	PA/PSf	1.25 ± 0.17	N.A.	N.A.	0.71 ± 0.14	17.25 ± 0.96	6.56 [b]	0.38±0.056	AL-DS	10 mM NaCl	0.5 M NaCl	[54]

	Membrane ID	Membrane structure	Membrane material	Membrane property				FO performance and testing conditions						Reference
				A (L/(m^2 h bar))	B_{NaCl} (L/(m^2 h))	B_{NaCl}/A (bar)	S (mm)	J_v (L/(m^2 h))	J_s (g/(m^2 h))	J_s/J_v (g/L)	Membrane orientation	Feed solution	Draw solution	
								9.03±0.44	2.89 [b]	0.32±0.12	AL-FS	10 mM NaCl	0.5 M NaCl	
55	xLbL3	LbL flat-sheet membrane	PAH-PSS/PAN	6.9 ± 1.6	N.A.	0.133 ± 0.018 [d]	N.A.	~60 [a]	6 [b]	~0.1 [a]	AL-DS	DI water	0.5 M $MgCl_2$	[55]
								~30 [a]	9 [b]	~0.3 [a]	AL-FS	DI water	0.5 M $MgCl_2$	
56	NC-FO	TFC flat-sheet membrane with nonwoven-fabric-reinforced nanofiber substrate	PA/PES/PET	1.65 ± 0.14	N.A.	N.A.	0.106 ± 0.008	~35 [a]	N.A.	N.A.	AL-DS	DI water	0.5 M NaCl	[56]
								~35 [a]	N.A.	N.A.	AL-FS	DI water	0.5 M NaCl	
57	3#LbL FO	LbL flat-sheet membrane	PAH-PSS/PAN	10.22 ± 2.34	3.46 ± 0.07 [d]	0.338 [d]	0.5 ± 0.2	31.7	46.65	1.48	AL-DS	DI water	1 M $MgCl_2$	[57]
								28.7	17.13	0.60	AL-FS	DI water	1 M $MgCl_2$	
58	TFC-2	TFC flat-sheet membrane	PA/PSf	1.78 ± 0.23	0.34 ± 0.07	0.20 ± 0.06	0.67 ± 0.17	20.5	5.9	0.29 [b]	AL-DS	10 mM NaCl	0.5 M NaCl	[58]
								12.0	4.9	0.41 [b]	AL-FS	10 mM NaCl	0.5 M NaCl	
59	TFC-FO	TFC flat-sheet membrane with nonwoven fabric	PA/PSf/PET	1.16 ± 0.06	0.47 ± 0.11	0.41 [b]	0.492 ± 0.038	~11 [a]	N.A.	N.A.	AL-FS	DI water	0.5 M NaCl	[59]

	Membrane ID	Membrane structure	Membrane material	Membrane property				FO performance and testing conditions						Reference
				A (L/(m^2 h bar))	B_{NaCl} (L/(m^2 h))	B_{NaCl}/A (bar)	S (mm)	J_v (L/(m^2 h))	J_s (g/(m^2 h))	J_s/J_v (g/L)	Membrane orientation	Feed solution	Draw solution	
60	RGO/CNT	Hollow fiber membrane	GO/PVB-CNT	2.11	0.051	0.024 [b]	0.202	22.6	1.6	0.07	AL-FS	DI water	0.5 M NaCl	[60]
61	100 kDa	TFC hollow fiber membrane	PA/PSf	~0.4 [c]	~0.02 [c]	0.05 [b]	0.725 ± 0.075	~29 [a]	3.3	0.11 [b]	AL-DS	DI water	1 M NaCl	[61]
								~10 [a]	2.1	0.21 [b]	AL-FS	DI water	1 M NaCl	
62	TFC-FO (HF-A)	TFC hollow fiber membrane	PA/PK	1.2 [c]	0.265 [c]	0.22 [b]	0.250	~41 [a]	~12 [a]	0.29 [b]	AL-DS	DI water	0.5 M NaCl	[62]
63	PES-hollow fiber	TFC hollow fiber membrane	PA/PES	2.21 ± 0.09	1.22 ± 0.05	0.55 [b]	1.09 ± 0.083	15.3 ± 1.3	N.A.	N.A.	AL-FS	DI water	0.5 M NaCl	[32]
64	DS#1.5	Double-skinned TFC hollow fiber membrane	PA/PES/PAH-PSS	2.64	0.14	0.05 [b]	N.A.	~40 [a]	4 [b]	~0.1 [a]	AL-DS	DI water	0.5 M NaCl	[63]
								~17 [a]	3.4 [b]	~0.2 [a]	AL-FS	DI water	0.5 M NaCl	
65	LBL-2I	LbL hollow fiber membrane	Poly(styrene sulfonate)-PAH-PDADMAC-PEI/PES	9.8	N.A.	N.A.	N.A.	73	4.38 [b]	0.06	AL-DS	DI water	0.5 M $MgCl_2$	[64]
66	LPR 100	TFC hollow fiber membrane	PA-aquaporin-incorporated	~8 [a]	~1.26 [a]	0.16 [b]	N.A.	55.2 ± 4.5	4.5 ± 0.2	0.08 [b]	AL-DS	DI water	0.5 M NaCl	[65]

	Membrane ID	Membrane structure	Membrane material	Membrane property				FO performance and testing conditions						Reference
				A (L/(m[2] h bar))	B_{NaCl} (L/(m[2] h))	B_{NaCl}/A (bar)	S (mm)	J_v (L/(m[2] h))	J_s (g/(m[2] h))	J_s/J_v (g/L)	Membrane orientation	Feed solution	Draw solution	
			proteoliposome/PES											
67	TFC-TB3	TFC hollow fiber membrane with tribore substrate	PA/matrimid	1.51	0.44	0.29 [b]	1.10	~22 [a]	~2.3 [a]	0.10 [b]	AL-DS	DI water	0.5 M NaCl	[66]
								~7 [a]	~1.5 [a]	0.21 [b]	AL-FS	DI water	0.5 M NaCl	
68	CTA TFC	TFC hollow fiber membrane	PA/CTA	0.85 ± 0.13	0.11 ± 0.02	0.13 [b]	0.236 ± 0.025	27	N.A.	N.A.	AL-DS	DI water	0.58 M NaCl	[67]
								13	N.A.	N.A.	AL-FS	DI water	0.58 M NaCl	
69	TFC 1.5 mol % sPPSU	TFC hollow fiber membrane	PA/PPSU-sPPSU	1.99 ± 0.02	0.0399 ± 0.002	0.02 [b]	0.163	49.39 ± 6.2	11.00 ± 1.36	0.22	AL-DS	DI water	0.5 M NaCl	[68]
								22.51 ± 2.3	5.49 ± 0.35	0.24	AL-FS	DI water	0.5 M NaCl	
70	DL-25K-d	Dual-layer hollow fiber membrane	Torlon® 4000T-MV-PEI-Poly(styrenesulfonate)-PAH-GA/PES	4.10	0.08 g/m[2] h [d]	N.A.	0.633	39.3	13.76 [b]	0.35	AL-DS	DI water	0.5 M $MgCl_2$	[69]
								20.8	6.45 [b]	0.31	AL-FS	DI water	0.5 M $MgCl_2$	
71	TFC-FO with PES_{water} supports	TFC hollow fiber membrane	PA/PES	1.18	0.135	0.11 [b]	0.219	25.6	3.2	0.13 [b]	AL-DS	DI water	0.5 M NaCl	[70]
								22.5	2.8	0.12 [b]	AL-FS	DI water	0.5 M NaCl	
72	TFC hollow		PA/PES	3.32	0.139	0.04 [b]	0.46	40.3	80.6 [b]	~2 [a]	AL-DS	10 mM NaCl	0.5 M NaCl	[71]

	Membrane ID	Membrane structure	Membrane material	Membrane property				FO performance and testing conditions						Reference
				A (L/(m^2 h bar))	B_{NaCl} (L/(m^2 h))	B_{NaCl}/A (bar)	*S* (mm)	J_v (L/(m^2 h))	J_s (g/(m^2 h))	J_s/J_v (g/L)	Membrane orientation	Feed solution	Draw solution	
	fiber membrane	TFC hollow fiber membrane						17.3	17.3 [b]	~1 [a]	AL-FS	10 mM NaCl	0.5 M NaCl	
73	#A-FO	TFC hollow fiber membrane	PA/PES	3.29	0.11	0.03 [b]	0.63 ± 0.02	47.7	3.5	0.07 [b]	AL-DS	DI water	0.5 M NaCl	[72]
							0.49 ± 0.06	18.6	2.0	0.11 [b]	AL-FS	DI water	0.5 M NaCl	
74	#B-FO	TFC hollow fiber membrane	PA/PES	2.22	0.20	0.09 [b]	0.595	32.2	~4 [a]	0.11	AL-DS	DI water	0.5 M NaCl	[73]
								14	1.75	0.13	AL-FS	DI water	0.5 M NaCl	

[a] Data was obtained from the figures in the references. [b] The value was calculated based on the data provided in references. [c] The value was determined by FO experiments. [d] The value was measured using $MgCl_2$ solution as feed.

Table S2. Abbreviations of membrane materials

Abbreviations	Membrane materials
AEPPS	N-aminoethyl piperazine propane sulfonate
CA	Cellulose acetate
CAP	Cellulose acetate propionate
CN/rGO	Reduced graphene oxide modified graphitic carbon nitride
CNT	Carbon nanotube
CTA	Cellulose triacetate
F-MWCNTs	Functionalized multi-walled carbon nanotubes
GO	Graphene oxide
HNT	Halloysite nanotube
IER-Na	Na type strong acid cation exchange resin
LDH	Layered double hydroxide
MOF	Metal–organic framework
PA	Polyamide
PAA	Poly(acrylic acid)
PAH	Poly(allylamine hydrochloride)
PAN	Polyacrylonitrile
PDA	Polydopamine
PDADMAC	Poly(diallyl-dimethylammonium chloride)
PE	Polyethylene
PEI	Poly(ethylene imine)
PES	Poly(ether sulfone)
PET	Poly(ethylene terephthalate)
PFSA	Perfluorosulfonic acid
PK	Polyketone
PPSU	Poly(phenylene sulfone)
PSf	Polysulfone
PSS	Poly(sodium 4-styrene-sulfonate)
PVA	Poly(vinyl alcohol)
PVB	Poly(vinyl butyral)
PVC	Poly(vinyl chloride)
PVDF	Poly(vinylidene fluoride)
SPEEK	Sulfonated poly(ether ether ketone)
SPPO	Sulfonated poly(phenylene oxide)
sPPSU	Sulfonated poly(phenylene sulfone)
SPSf	Sulfonated polysulfone
ZSCSNPs	ZnO-SiO2 core-shell nanoparticles

References

1. Saeedi-Jurkuyeh, A.; Jafari, A.J.; Kalantary, R.R.; Esrafili, A. A novel synthetic thin-film nanocomposite forward osmosis membrane modified by graphene oxide and polyethylene glycol for heavy metals removal from aqueous solutions. *Reactive and Functional Polymers* **2020**, *146*, 104397.
2. Pang, J.; Kang, Z.; Wang, R.; Xu, B.; Nie, X.; Fan, L.; Zhang, F.; Du, X.; Feng, S.; Sun, D. Exploring the sandwich antibacterial membranes based on UiO-66/graphene oxide for forward osmosis performance. *Carbon* **2019**, *144*, 321-332.
3. Chiao, Y.-H.; Sengupta, A.; Chen, S.-T.; Huang, S.-H.; Hu, C.-C.; Hung, W.-S.; Chang, Y.; Qian, X.; Wickramasinghe, S.R.; Lee, K.-R., et al. Zwitterion augmented polyamide membrane for improved forward osmosis performance with significant antifouling characteristics. *Separation and Purification Technology* **2019**, *212*, 316-325.

4. Kwon, S.J.; Park, S.-H.; Shin, M.G.; Park, M.S.; Park, K.; Hong, S.; Park, H.; Park, Y.-I.; Lee, J.-H. Fabrication of high performance and durable forward osmosis membranes using mussel-inspired polydopamine-modified polyethylene supports. *Journal of Membrane Science* **2019**, *584*, 89-99.
5. Sirinupong, T.; Youravong, W.; Tirawat, D.; Lau, W.J.; Lai, G.S.; Ismail, A.F. Synthesis and characterization of thin film composite membranes made of PSF-TiO2/GO nanocomposite substrate for forward osmosis applications. *Arabian Journal of Chemistry* **2018**, *11*, 1144-1153.
6. Rastgar, M.; Shakeri, A.; Bozorg, A.; Salehi, H.; Saadattalab, V. Highly-efficient forward osmosis membrane tailored by magnetically responsive graphene oxide/Fe3O4 nanohybrid. *Applied Surface Science* **2018**, *441*, 923-935.
7. Rezaei-DashtArzhandi, M.; Sarrafzadeh, M.H.; Goh, P.S.; Lau, W.J.; Ismail, A.F.; Mohamed, M.A. Development of novel thin film nanocomposite forward osmosis membranes containing halloysite/graphitic carbon nitride nanoparticles towards enhanced desalination performance. *Desalination* **2018**, *447*, 18-28.
8. Kwon, S.J.; Park, S.-H.; Park, M.S.; Lee, J.S.; Lee, J.-H. Highly permeable and mechanically durable forward osmosis membranes prepared using polyethylene lithium ion battery separators. *J. Membr. Sci.* **2017**, *544*, 213-220.
9. Zuo, H.-R.; Lu, H.; Cao, G.-P.; Wang, M.; Wang, Y.-Y.; Liu, J.-M. Ion exchange resin blended membrane: Enhanced water transfer and retained salt rejection for forward osmosis. *Desalination* **2017**, *421*, 12-22.
10. Rastgar, M.; Shakeri, A.; Bozorg, A.; Salehi, H.; Saadattalab, V. Impact of nanoparticles surface characteristics on pore structure and performance of forward osmosis membranes. *Desalination* **2017**, *421*, 179-189.
11. Tian, E.; Wang, X.; Zhao, Y.; Ren, Y. Middle support layer formation and structure in relation to performance of three-tier thin film composite forward osmosis membrane. *Desalination* **2017**, *421*, 190-201.
12. Pardeshi, P.M.; Mungray, A.K.; Mungray, A.A. Polyvinyl chloride and layered double hydroxide composite as a novel substrate material for the forward osmosis membrane. *Desalination* **2017**, *421*, 149-159.
13. Zhang, X.; Shen, L.; Lang, W.-Z.; Wang, Y. Improved performance of thin-film composite membrane with PVDF/PFSA substrate for forward osmosis process. *J. Membr. Sci.* **2017**, *535*, 188-199.
14. Zirehpour, A.; Rahimpour, A.; Ulbricht, M. Nano-sized metal organic framework to improve the structural properties and desalination performance of thin film composite forward osmosis membrane. *J. Membr. Sci.* **2017**, *531*, 59-67.
15. Liang, H.-Q.; Hung, W.-S.; Yu, H.-H.; Hu, C.-C.; Lee, K.-R.; Lai, J.-Y.; Xu, Z.-K. Forward osmosis membranes with unprecedented water flux. *J. Membr. Sci.* **2017**, *529*, 47-54.
16. Chen, G.; Liu, R.; Shon, H.K.; Wang, Y.; Song, J.; Li, X.-M.; He, T. Open porous hydrophilic supported thin-film composite forward osmosis membrane via co-casting for treatment of high-salinity wastewater. *Desalination* **2017**, *405*, 76-84.
17. Ma, D.; Peh, S.B.; Han, G.; Chen, S.B. Thin-Film Nanocomposite (TFN) Membranes Incorporated with Super-Hydrophilic Metal–Organic Framework (MOF) UiO-66: Toward Enhancement of Water Flux and Salt Rejection. *ACS Appl. Mater. Interfaces* **2017**, *9*, 7523-7534.
18. Li, X.; Loh, C.H.; Wang, R.; Widjajanti, W.; Torres, J. Fabrication of a robust high-performance FO membrane by optimizing substrate structure and incorporating aquaporin into selective layer. *J. Membr. Sci.* **2017**, *525*, 257-268.
19. Lim, S.; Park, M.J.; Phuntsho, S.; Tijing, L.D.; Nisola, G.M.; Shim, W.-G.; Chung, W.-J.; Shon, H.K. Dual-layered nanocomposite substrate membrane based on polysulfone/graphene oxide for mitigating internal concentration polarization in forward osmosis. *Polymer* **2017**, *110*, 36-48.
20. Ding, W.; Li, Y.; Bao, M.; Zhang, J.; Zhang, C.; Lu, J. Highly permeable and stable forward osmosis (FO) membrane based on the incorporation of Al2O3 nanoparticles into both substrate and polyamide active layer. *RSC Advances* **2017**, *7*, 40311-40320.
21. Pan, S.-F.; Dong, Y.; Zheng, Y.-M.; Zhong, L.-B.; Yuan, Z.-H. Self-sustained hydrophilic nanofiber thin film composite forward osmosis membranes: Preparation, characterization and application for simulated antibiotic wastewater treatment. *Journal of Membrane Science* **2017**, *523*, 205-215.
22. Yasukawa, M.; Mishima, S.; Tanaka, Y.; Takahashi, T.; Matsuyama, H. Thin-film composite forward osmosis membrane with high water flux and high pressure resistance using a thicker void-free polyketone porous support. *Desalination* **2017**, *402*, 1-9.
23. Xiong, S.; Zuo, J.; Ma, Y.G.; Liu, L.; Wu, H.; Wang, Y. Novel thin film composite forward osmosis membrane of enhanced water flux and anti-fouling property with N-[3-(trimethoxysilyl) propyl] ethylenediamine incorporated. *J. Membr. Sci.* **2016**, *520*, 400-414.
24. Zhang, X.; Tian, J.; Ren, Z.; Shi, W.; Zhang, Z.; Xu, Y.; Gao, S.; Cui, F. High performance thin-film composite (TFC) forward osmosis (FO) membrane fabricated on novel hydrophilic disulfonated poly(arylene ether sulfone) multiblock copolymer/polysulfone substrate. *J. Membr. Sci.* **2016**, *520*, 529-539.
25. Duong, P.H.H.; Nunes, S.P.; Chung, T.-S. Dual-skinned polyamide/poly(vinylidene fluoride)/cellulose acetate membranes with embedded woven. *J. Membr. Sci.* **2016**, *520*, 840-849.
26. Bui, N.-N.; McCutcheon, J.R. Nanoparticle-embedded nanofibers in highly permselective thin-film nanocomposite membranes for forward osmosis. *J. Membr. Sci.* **2016**, *518*, 338-346.
27. Khorshidi, B.; Bhinder, A.; Thundat, T.; Pernitsky, D.; Sadrzadeh, M. Developing high throughput thin film composite polyamide membranes for forward osmosis treatment of SAGD produced water. *Journal of Membrane Science* **2016**, *511*, 29-39.

28. Song, X.; Wang, L.; Mao, L.; Wang, Z. Nanocomposite Membrane with Different Carbon Nanotubes Location for Nanofiltration and Forward Osmosis Applications. *ACS Sustainable Chem. Eng.* **2016**, *4*, 2990-2997.
29. Lu, P.; Liang, S.; Qiu, L.; Gao, Y.; Wang, Q. Thin film nanocomposite forward osmosis membranes based on layered double hydroxide nanoparticles blended substrates. *J. Membr. Sci.* **2016**, *504*, 196-205.
30. Han, G.; Zhao, B.; Fu, F.; Chung, T.-S.; Weber, M.; Staudt, C.; Maletzko, C. High performance thin-film composite membranes with mesh-reinforced hydrophilic sulfonated polyphenylenesulfone (sPPSU) substrates for osmotically driven processes. *J. Membr. Sci.* **2016**, *502*, 84-93.
31. Huang, L.; Arena, J.T.; McCutcheon, J.R. Surface modified PVDF nanofiber supported thin film composite membranes for forward osmosis. *J. Membr. Sci.* **2016**, *499*, 352-360.
32. Zhang, M.; Liu, R.; Wang, Z.; Zhao, B.; Song, J.; Park, M.J.; Shon, H.K.; Li, X.-M.; He, T. Dehydration of forward osmosis membranes in treating high salinity wastewaters: Performance and implications. *J. Membr. Sci.* **2016**, *498*, 365-373.
33. Liu, Q.; Li, J.; Zhou, Z.; Xie, J.; Lee, J.Y. Hydrophilic Mineral Coating of Membrane Substrate for Reducing Internal Concentration Polarization (ICP) in Forward Osmosis. *Sci. Rep.* **2016**, *6*.
34. Ghanbari, M.; Emadzadeh, D.; Lau, W.J.; Riazi, H.; Almasi, D.; Ismail, A.F. Minimizing structural parameter of thin film composite forward osmosis membranes using polysulfone/halloysite nanotubes as membrane substrates. *Desalination* **2016**, *377*, 152-162.
35. Wei, R.; Zhang, S.; Cui, Y.; Ong, R.C.; Chung, T.-S.; Helmer, B.J.; de Wit, J.S. Highly permeable forward osmosis (FO) membranes for high osmotic pressure but viscous draw solutes. *J. Membr. Sci.* **2015**, *496*, 132-141.
36. Park, M.J.; Phuntsho, S.; He, T.; Nisola, G.M.; Tijing, L.D.; Li, X.-M.; Chen, G.; Chung, W.-J.; Shon, H.K. Graphene oxide incorporated polysulfone substrate for the fabrication of flat-sheet thin-film composite forward osmosis membranes. *J. Membr. Sci.* **2015**, *493*, 496-507.
37. Lee, J.-Y.; She, Q.; Huo, F.; Tang, C.Y. Metal–organic framework-based porous matrix membranes for improving mass transfer in forward osmosis membranes. *J. Membr. Sci.* **2015**, *492*, 392-399.
38. Qin, D.; Liu, Z.; Delai Sun, D.; Song, X.; Bai, H. A new nanocomposite forward osmosis membrane custom-designed for treating shale gas wastewater. *Sci. Rep.* **2015**, *5*.
39. Kwon, S.-B.; Lee, J.S.; Kwon, S.J.; Yun, S.-T.; Lee, S.; Lee, J.-H. Molecular layer-by-layer assembled forward osmosis membranes. *J. Membr. Sci.* **2015**, *488*, 111-120.
40. Xiao, P.; Nghiem, L.D.; Yin, Y.; Li, X.M.; Zhang, M.; Chen, G.; Song, J.; He, T. A sacrificial-layer approach to fabricate polysulfone support for forward osmosis thin-film composite membranes with reduced internal concentration polarisation. *Journal of Membrane Science* **2015**, *481*, 106-114.
41. Wang, Y.; Ou, R.; Wang, H.; Xu, T. Graphene oxide modified graphitic carbon nitride as a modifier for thin film composite forward osmosis membrane. *Journal of Membrane Science* **2015**, *475*, 281-289.
42. Liu, X.; Ng, H.Y. Double-blade casting technique for optimizing substrate membrane in thin-film composite forward osmosis membrane fabrication. *J. Membr. Sci.* **2014**, *469*, 12-126.
43. Stillman, D.; Krupp, L.; La, Y.H. Mesh-reinforced thin film composite membranes for forward osmosis applications: The structure-performance relationship. *Journal of Membrane Science* **2014**, *468*, 308-316.
44. Ong, R.C.; Chung, T.S.; de Wit, J.; Helmer, B.J. Novel cellulose ester substrates for high performance flat-sheet thin-film composite (TFC) forward osmosis (FO) membranes. *Journal of Membrane Science* **2014**, *473*, 63-71.
45. Zhou, Z.; Lee, J.Y.; Chung, T.S. Thin film composite forward-osmosis membranes with enhanced internal osmotic pressure for internal concentration polarization reduction. *Chemical Engineering Journal* **2014**, *249*, 236-245.
46. Puguan, J.M.C.; Kim, H.S.; Lee, K.J.; Kim, H. Low internal concentration polarization in forward osmosis membranes with hydrophilic crosslinked PVA nanofibers as porous support layer. *Desalination* **2014**, *336*, 24-31.
47. Emadzadeh, D.; Lau, W.J.; Ismail, A.F. Synthesis of thin film nanocomposite forward osmosis membrane with enhancement in water flux without sacrificing salt rejection. *Desalination* **2013**, *330*, 90-99.
48. You, S.; Tang, C.; Yu, C.; Wang, X.; Zhang, J.; Han, J.; Gan, Y.; Ren, N. Forward osmosis with a novel thin-film inorganic membrane. *Environmental Science and Technology* **2013**, *47*, 8733-8742.
49. Amini, M.; Jahanshahi, M.; Rahimpour, A. Synthesis of novel thin film nanocomposite (TFN) forward osmosis membranes using functionalized multi-walled carbon nanotubes. *Journal of Membrane Science* **2013**, *435*, 233-241.
50. Widjojo, N.; Chung, T.S.; Weber, M.; Maletzko, C.; Warzelhan, V. A sulfonated polyphenylenesulfone (sPPSU) as the supporting substrate in thin film composite (TFC) membranes with enhanced performance for forward osmosis (FO). *Chem. Eng.J.* **2013**, *220*, 15-23.
51. Wei, J.; Qiu, C.; Wang, Y.N.; Wang, R.; Tang, C.Y. Comparison of NF-like and RO-like thin film composite osmotically-driven membranes-Implications for membrane selection and process optimization. *Journal of Membrane Science* **2013**, *427*, 460-471.
52. Tiraferri, A.; Kang, Y.; Giannelis, E.P.; Elimelech, M. Superhydrophilic thin-film composite forward osmosis membranes for organic fouling control: Fouling behavior and antifouling mechanisms. *Environmental Science and Technology* **2012**, *46*, 11135-11144.
53. Ma, N.; Wei, J.; Liao, R.; Tang, C.Y. Zeolite-polyamide thin film nanocomposite membranes: Towards enhanced performance for forward osmosis. *J. Membr. Sci.* **2012**, *405-406*, 149-157.

54. Wei, J.; Liu, X.; Qiu, C.; Wang, R.; Tang, C.Y. Influence of monomer concentrations on the performance of polyamide-based thin film composite forward osmosis membranes. *J. Membr. Sci.* **2011**, *381*, 110-117.
55. Qiu, C.; Qi, S.; Tang, C.Y. Synthesis of high flux forward osmosis membranes by chemically crosslinked layer-by-layer polyelectrolytes. *J. Membr. Sci.* **2011**, *381*, 74-80.
56. Song, X.; Liu, Z.; Sun, D.D. Nano gives the answer: Breaking the bottleneck of internal concentration polarization with a nanofiber composite forward osmosis membrane for a high water production rate. *Adv. Mater.* **2011**, *23*, 3256-3260.
57. Saren, Q.; Qiu, C.Q.; Tang, C.Y. Synthesis and characterization of novel forward osmosis membranes based on layer-by-layer assembly. *Environ. Sci. Technol.* **2011**, *45*, 5201-5208.
58. Wei, J.; Qiu, C.; Tang, C.Y.; Wang, R.; Fane, A.G. Synthesis and characterization of flat-sheet thin film composite forward osmosis membranes. *Journal of Membrane Science* **2011**, *372*, 292-302.
59. Yip, N.Y.; Tiraferri, A.; Phillip, W.A.; Schiffman, J.D.; Elimelech, M. High performance thin-film composite forward osmosis membrane. *Environ. Sci. Technol.* **2010**, *44*, 3812-3818.
60. Fan, X.; Liu, Y.; Quan, X. A novel reduced graphene oxide/carbon nanotube hollow fiber membrane with high forward osmosis performance. *Desalination* **2019**, *451*, 117-124.
61. Ren, J.; Chowdhury, M.R.; Qi, J.; Xia, L.; Huey, B.D.; McCutcheon, J.R. Relating osmotic performance of thin film composite hollow fiber membranes to support layer surface pore size. *J. Membr. Sci.* **2017**, *540*, 344-353.
62. Shibuya, M.; Yasukawa, M.; Mishima, S.; Tanaka, Y.; Takahashi, T.; Matsuyama, H. A thin-film composite-hollow fiber forward osmosis membrane with a polyketone hollow fiber membrane as a support. *Desalination* **2017**, *402*, 33-41.
63. Fang, W.; Liu, C.; Shi, L.; Wang, R. Composite forward osmosis hollow fiber membranes: Integration of RO- and NF-like selective layers for enhanced organic fouling resistance. *Journal of Membrane Science* **2015**, *492*, 147-155.
64. Liu, C.; Shi, L.; Wang, R. Enhanced hollow fiber membrane performance via semi-dynamic layer-by-layer polyelectrolyte inner surface deposition for nanofiltration and forward osmosis applications. *Reactive and Functional Polymers* **2015**, *86*, 154-160.
65. Li, X.; Chou, S.; Wang, R.; Shi, L.; Fang, W.; Chaitra, G.; Tang, C.Y.; Torres, J.; Hu, X.; Fane, A.G. Nature gives the best solution for desalination: Aquaporin-based hollow fiber composite membrane with superior performance. *J. Membr. Sci.* **2015**, *494*, 68-77.
66. Luo, L.; Wang, P.; Zhang, S.; Han, G.; Chung, T.S. Novel thin-film composite tri-bore hollow fiber membrane fabrication for forward osmosis. *Journal of Membrane Science* **2014**, *461*, 28-38.
67. Zhang, S.; Wang, P.; Fu, X.; Chung, T.S. Sustainable water recovery from oily wastewater via forward osmosis-membrane distillation (FO-MD). *Water Research* **2014**, *52*, 112-121.
68. Zhong, P.; Fu, X.; Chung, T.S.; Weber, M.; Maletzko, C. Development of thin-film composite forward osmosis hollow fiber membranes using direct sulfonated polyphenylenesulfone (sPPSU) as membrane substrates. *Environ. Sci. Technol.* **2013**, *47*, 7430-7436.
69. Setiawan, L.; Wang, R.; Tan, S.; Shi, L.; Fane, A.G. Fabrication of poly(amide-imide)-polyethersulfone dual layer hollow fiber membranes applied in forward osmosis by combined polyelectrolyte cross-linking and depositions. *Desalination* **2013**, *312*, 99-106.
70. Sukitpaneenit, P.; Chung, T.S. High performance thin-film composite forward osmosis hollow fiber membranes with macrovoid-free and highly porous structure for sustainable water production. *Environ. Sci. Technol.* **2012**, *46*, 7358-7365.
71. Chou, S.; Wang, R.; Shi, L.; She, Q.; Tang, C.; Fane, A.G. Thin-film composite hollow fiber membranes for pressure retarded osmosis (PRO) process with high power density. *Journal of Membrane Science* **2012**, *389*, 25-33.
72. Shi, L.; Chou, S.R.; Wang, R.; Fang, W.X.; Tang, C.Y.; Fane, A.G. Effect of substrate structure on the performance of thin-film composite forward osmosis hollow fiber membranes. *J. Membr. Sci.* **2011**, *382*, 116-123.
73. Wang, R.; Shi, L.; Tang, C.Y.; Chou, S.; Qiu, C.; Fane, A.G. Characterization of novel forward osmosis hollow fiber membranes. *Journal of Membrane Science* **2010**, *355*, 158-167.
